# Comparison of different methods for isolating CD8^+^ T lymphocyte‐derived extracellular vesicles and supramolecular attack particles

**DOI:** 10.1002/jex2.74

**Published:** 2023-03-13

**Authors:** Ashwin K. Jainarayanan, Jesusa Capera, Pablo F. Céspedes, Mariana Conceição, Mirudula Elanchezhian, Tom Thomas, Scott Bonner, Salvatore Valvo, Elke Kurz, Ranjeet Singh Mahla, Georgina Berridge, Svenja Hester, Roman Fischer, Lynn B. Dustin, Matthew J. A. Wood, Michael L. Dustin

**Affiliations:** ^1^ Kennedy Institute of Rheumatology, Nuffield Department of Orthopedics, Rheumatology and, Musculoskeletal Sciences University of Oxford Oxford UK; ^2^ Interdisciplinary Bioscience Doctoral Training Program and Exeter College University of Oxford Oxford UK; ^3^ Department of Paediatrics University of Oxford Oxford UK; ^4^ Department of Biological Sciences Indian Institute of Science Education and Research Mohali India; ^5^ Translational Gastroenterology Unit University of Oxford Oxford UK; ^6^ Wellcome Centre for Human Genetics University of Oxford Oxford UK; ^7^ Target Discovery Institute, Centre for Medicines Discovery, Nuffield Department of Medicine University of Oxford Oxford UK; ^8^ MDUK Oxford Neuromuscular Centre University of Oxford Oxford UK; ^9^ Oxford‐Harrington Rare Disease Centre University of Oxford Oxford UK

**Keywords:** CD8^+^ T cells, extracellular vesicles, proteomics and lipidomics, size exclusion liquid chromatography

## Abstract

CD8^+^ T lymphocytes play vital roles in killing infected or deranged host cells, recruiting innate immune cells, and regulating other aspects of immune responses. Like any other cell, CD8^+^ T cells also produce extracellular particles. These include extracellular vesicles (EVs) and non‐vesicular extracellular particles (NVEPs). T cell‐derived EVs are proposed to mediate cell‐to‐cell signalling, especially in the context of inflammatory responses, autoimmunity, and infectious diseases. CD8^+^ T cells also produce supramolecular attack particles (SMAPs), which are in the same size range as EVs and mediate a component of T cell mediated killing. The isolation technique selected will have a profound effect on yield, purity, biochemical properties and function of T cell‐derived particles; making it important to directly compare different approaches. In this study, we compared commonly used techniques (membrane spin filtration, ultracentrifugation, or size exclusion liquid chromatography) to isolate particles from activated human CD8^+^ T cells and validated our results by single‐particle methods, including nanoparticle tracking analysis, flow cytometry, electron microscopy and super‐resolution microscopy of the purified sample as well as bulk proteomics and lipidomics analyses to evaluate the quality and nature of enriched T cell‐derived particles. Our results show that there is a trade‐off between the yield and the quality of T cell‐derived particles. Furthermore, the protein and lipid composition of the particles is dramatically impacted by the isolation technique applied. We conclude that from the techniques evaluated, size exclusion liquid chromatography offers the highest quality of T cell derived EVs and SMAPs with acceptable yields for compositional and functional studies.

## 1 INTRODUCTION

T lymphocytes coordinate numerous innate and adaptive immunity components and defend against many simultaneous attacks across the body to maintain homeostasis. This requires coordinated responses among cell networks to facilitate pathogen control while maintaining self‐tolerance and tissue functionality. Breakdown of this regulatory intercellular crosstalk can lead to immunopathology (Uhl & Gérard, 2020). T cells communicate with neighbouring cells directly by forming physical contacts including immunological synapse (Basu & Huse, 2017) and membrane nanotubes (Sowinski et al., 2008), and indirectly through the secretion of soluble factors like cytokines and chemokines in the local environment, allowing them to translate and coordinate local interactions into tissue‐wide behaviour (Uhl & Gerard, 2020). Some of the secreted proteins are released in EVs and core‐shell particles referred to as supramolecular attack particles (SMAPs) (Balint et al., 2020). EVs enable immune cells to share cytoplasmic and membrane‐anchored messages with each other and their cellular microenvironment to support coordination (Céspedes et al., 2022; Choudhuri et al., 2014; Okoye et al., 2014; Saliba et al., 2019). SMAPs were discovered in association with cell mediated killing, but NVEPs may have broader roles in T cell biology.

EVs are nanometre to micrometre‐sized phospholipid membrane‐bound compartments, which, based on their origins, can be classified into various subtypes such as exosomes, microvesicles/ectosomes, and apoptotic bodies (Raposo & Stoorvogel, 2013; Théry et al., 2018; Willms et al., 2018). A general characteristic is that the contents of EVs are generally cell cytoplasm, the inner leaflet of the phospholipid bilayers corresponds to the cytoplasmic leaflet and the outer leaflet to the extracellular surface. EVs mediate intercellular communication via horizontal transfer of proteins, nucleic acids and lipids, being involved in multifaceted physiological and pathological activities (Gomes et al., 2015; Skog et al., 2008; Valadi et al., 2007). EVs from different T cell subtypes have been observed to have different compositions and to play immunoregulatory roles (Li et al., 2016; Wu et al., 2019; Yang et al., 2019; Zhang et al., 2011), but contamination by NVEP's may have also influenced these results.

The use of EVs as therapeutic agents, particularly EVs released from T cells, is gaining attention. The recent discovery of SMAPs with their own value in therapeutic settings has also raised interest in tracking SMAPs markers within EV preparations. Understanding how different isolation techniques affect the yield, purity, and biochemical properties of T cell‐released particles, including the relative levels of and the level of EVs and SMAPs is critical in taking this modality from bench to bedside. It has been reported that proteomic profiles of particles from the same cell source significantly vary according to the isolation method (Yáñez‐Mó et al., 2015). Apart from this, there are multiple limitations related to the isolation of particles from T cells, such as specific conditions for T cell growth, the low or uncharacterized net release rate of EVs and NVEPs (Brennan et al., 2020; Szatanek et al., 2015).

Techniques commonly used for EV isolation from cell cultures, namely, differential ultracentrifugation (UC), size exclusion liquid chromatography (SELC), membrane spin filtration (MSF), sucrose gradient centrifugation or PEG precipitation, take advantage of EV intrinsic properties such as size, charge and density (Brennan et al., 2020; Liangsupree et al., 2021; Lee et al., 2019). Nevertheless, they have their limitations concerning yield, cost and purity (Konoshenko et al., 2018). It is generally unknown if any of these methods have differential effects on SMAP/NVEP enrichment. For instance, though precipitation using PEG/ sodium acetate/protamine allows EV isolation from large culture volumes and does not require expensive reagents or equipment, the overall disadvantages include contamination with NVEPs such as lipoproteins, nucleoproteins, and other particles, as well as retention of some chemicals or polymers (when these are used to engineer EVs) (Konoshenko et al., 2018). To reduce contamination, several microfluidic‐based EV isolation techniques such as deterministic lateral displacement (DLD) pillar array (Wunsch et al., 2016), field flow fractionation (Zhang & Lyden, 2019), carbon nanotube arrays (Yeh et al., 2020), and λ‐DNA mediated viscoelastic microfluidic system (Liu et al., 2019) have been recently developed. However, the cost and complexity of these techniques represent a significant barrier to their widespread use (Konoshenko et al., 2018).

Bind‐elute size exclusion chromatography (BE‐SELC) is a more recent method for EV purification, which combines characteristics of SELC with ion‐exchange and hydrophobic interaction chromatography for components smaller than the resin's exclusion limit (Corso et al., 2017; Nordin et al., 2019; Onódi et al., 2018). However, low yield and impurities were present after using density gradient ultracentrifugation followed by BE‐SELC for EV isolation from blood plasma (Onódi et al., 2018). Recently, bead‐supported lipid bilayers (BSLBs) have been used as an elegant affinity‐based purification technology to study a specialized population of T cell EVs termed trans‐synaptic vesicles that are directly budded into synaptic spaces between cells or through directed release from multivesicular bodies (Céspedes et al., 2022). However, the beads also capture SMAPs and the approach is only applicable to EVs that are transferred through cell‐cell contact (Céspedes et al., 2022). Besides these, several EV isolation techniques can be performed in combination to improve EV quality (Okoye et al., 2014; Onódi et al., 2018; Tung et al., 2018). However, choosing a combination of separation techniques needs crucial attention to ensure the best results since the cost, time, purity, and yield must also be considered.

Here, we compare techniques that are commonly used to isolate EVs from other cell types: ultracentrifugation (UC), membrane spin filtration (MSF) and size‐exclusion liquid chromatography (SELC), which were chosen explicitly due to their wide applicability and reported impact on particle composition. We validated our results by single‐particle methods such as nanoparticle tracking analysis (NTA), electron microscopy (EM) and direct STochastic Optical Reconstruction Microscopy (dSTORM), as well as performing bulk proteomics and lipidomics to evaluate the quality and composition of the differently enriched T cell EVs. SMAPs can co‐isolate with EVs and therefore we have included SMAP markers in our analysis, to compare the fractionation behaviour of SMAPs using different isolation methods.

## 2 METHODS AND MATERIALS

### 2.1 T cell culture and expansion

Complete RPMI 1640 medium was prepared with 10% foetal bovine (exosome‐free) serum (heat‐inactivated), 100 μM non‐essential amino acids, 10 mM HEPES, 2 mM L‐glutamine, 1 mM sodium pyruvate, 100U/mL of penicillin and 100 μg/mL of streptomycin.

### 2.2 Isolation and purification of conventional CD8^+^ T cells

Human T cells were isolated from the leukoreduction system (LRS)‐concentrated peripheral blood using negative selection to leave untouched CD8^+^ T cells (RosetteSep, StemCell Technologies, #15023, #15063). Recovered CD8^+^ T cells were stimulated with Human T‐activator CD3/CD28 Dynabeads (ThermoFisher) at a bead‐to‐cell ratio of 1:1 and 100 U/mL of IL‐2. After 48 h of activation, Human T‐activator CD3/CD28 Dynabeads were removed, and the CD8^+^ T cells were maintained for 24 h in complete RPMI‐1640 media supplemented with 100 U/mL of IL‐2 at ∼2 × 10^6^ cells/mL before subjecting them to the downstream EV isolation processes.

For all the techniques described below, the T cell culture in different release media, as detailed in results, were first subjected to 300 g centrifugation twice for 5 min, and the supernatant retained to eliminate any cells and cell debris present in the media, followed by a 4000 g centrifugation for 10 min and the purified supernatant retained to eliminate bigger vesicles including some apoptotic bodies. All isolation technologies were tested from equivalent portions of the same purified supernatant (15 mL). The different replicates belong to three different donors, which each has been screened through all isolation technologies independently.

### 2.3 Membrane spin filtration

The purified supernatant was concentrated using 100 kDa molecular weight cut‐off Amicon centrifugal filter units (Merck Millipore, Billerica, MA, USA) at 4000 g to yield 5 mL of concentrate. The concentrate was washed with PBS thrice at room temperature (RT), and was further concentrated using 10 kDa MWCO pore size ultracel‐10 membranes (Merck Millipore) at 4000 g to yield 2 mL of a concentrated EV rich fraction.

### 2.4 Ultracentrifugation

The purified supernatant was centrifuged at 120,000 g for 4 h at 4°C, the supernatant was discarded and pellet was resuspended in 1 mL of PBS. In the case of re‐UC, the pellet was resuspended in 5 mL of PBS was re‐subjected to another round of UC as above, the supernatant discarded and the UC particles resuspended in 500 μL of PBS.

### 2.5 Size‐exclusion liquid chromatography

The purified supernatant was first concentrated using 100 kDa molecular weight cut‐off Amicon centrifugal filter units from 15 mL to 2 mL and subsequently loaded on a Sepharose 4 Fast Flow resin column (10 mm × 300 mm; GE Healthcare, Little Chalfont, UK), connected to the ÄKTA pure system (GE Healthcare) and eluted at 0.5 mL/min flow rate using PBS as the eluent. Chromatogram was recorded using absorbance at 280 nm. 0.5 mL fractions were collected, and SELC EV‐containing fractions were pooled and concentrated to 2 mL volume using 10 kDa molecular weight cut‐off Amicon centrifugal filter units.

### 2.6 Transmission electron microscopy

Negative stanning of vesicles preparation for transmission electron microscopy was performed as described elsewhere (Harris, 2007). In brief, carbon support 3 mm copper grids of 300 mesh were plasma treated with Leica EM ACE200 Vacuum Coater for 20 s. A 10 μL of vesicle preparation were deposited on it and incubated for 5 min at RT. The excess sample was absorbed with Whatman Type 1 filter paper. Prepared sample was dried for 10 min and analysed by 120 KV Tecnai 12 TEM fitted Gatan OneView CMOS camera. Vesicles were imaged at 29,000× magnification.

### 2.7 Density gradient ultracentrifugation (DGUC)

Cell culture purified supernatant was concentrated by spin filtration and mixed with 9 mL of 60% iodixanol solution (OptiPrep, Sigma), and slowly layered at bottom of a 38‐mL Ultra‐Clear tube (Beckman Coulter). Low density solution was prepared by dilution with homogenization buffer (10 mM Tris‐HCl, 250 mM sucrose, and 1 mM EDTA, pH 7.4) yielding final volume by volume iodixanol concentration of 30%, 23%, and 18%. The iodixanol layers of 30% (9 mL), 23% (6 mL), and 18% (6 mL) carefully added. These gradients were ultracentrifuged using swinging‐bucket SW 32 Ti rotor (Beckman Coulter) at 150,000×g for 16 h, at 4°C. Ultracentrifugation fractions were carefully isolated and diluted with PBS. Iodixanol contaminants were removed by successive ultra‐centrifugation as described elsewhere (Strobel et al., 2015). Finally, the DGUC particles were concentrated by membrane spin filtration.

### 2.8 Western blotting

Whole cell lysates (WCL) were prepared by resuspending cell pellets in RIPA lysis buffer (Thermo Fisher Scientific, #89901) containing a Protease/Phosphatase inhibitor (PI) cocktail (Cell Signaling Technology (CST); #5872) to a final concentration of 2 × 10^7^ cells/mL. After sonication at 4°C (10 cycles of 30 s on/30 s off), WCL were centrifuged at 10,000 g for 10 min at 4°C to remove cell debris, and the supernatants were collected. BCA was performed to determine the protein concentrations, and then the samples were mixed with loading solution and denatured at 95°C for 10 min. In the case of particles, isolated vesicles were subjected to RIPA and PI treatment, the protein concentrations were measured using micro‐BCA, and equal amounts of proteins across EV samples were loaded. Samples were resolved using 4%–15% Mini‐PROTEAN SDS‐PAGE gel (Bio‐Rad; #4561084), transferred to 0.45 μm nitrocellulose membranes (Bio‐Rad, #1620115), blocked and incubated with the following primary antibodies: rabbit anti‐TSG101 clone EPR7130B (#ab125011), rabbit anti‐APOA2 clone EP2912 (#ab119990), rabbit anti‐ALB clone EPR20195 (#ab207327), mouse anti‐LMNA clone 133A2 (#ab ab8980), rabbit anti‐HIST1H1.0 clone EPR6537 (#ab 125027), rabbit anti‐calnexin clone C5C9 (CST#2679), mouse anti‐ALIX clone 3A9 (CST#2171), rabbit anti‐GZMB (CST#4275), mouse anti‐PRF1 clone F‐1 (sc‐136994), rabbit anti‐TSP1 clone D7E5F (CST#37879), mouse anti‐CD81 clone M38 (Thermo Fisher Scientific#10630D), rabbit anti‐CD3 zeta clone EP286Y (#ab40804). Then, membranes were incubated with IRDye® 680RD donkey anti‐mouse IgG (H+L; LI‐COR, #926‐68072) and IRDye 800CW donkey anti‐rabbit IgG (H+L; LI‐COR, #925‐32213) secondary antibodies following manufacturer guidelines. After four washes, membranes were imaged and analysed using the Odyssey® CLx Near‐ Infrared detection system equipped with the Image Studio Lite quantification software (LICOR, Lincoln, NE).

### 2.9 Nanoparticle tracking analysis (NTA)

The isolated particles were diluted in PBS so that the concentrations detected would be between 5 × 10^8^ to 10^9^ particles per mL. The instrument used for NTA was the Nanosight NS500 (Malvern Instruments Ltd), together with the NTA 3.2 software, set on light scattering mode. Videos for NTA were captured for 60 s, three sequential replicates per sample were obtained, and the three recordings were processed and averaged to determine the mean size and concentration of the particles.

For the tracking analysis, TrackMate plugin from ImageJ was used (Tinevez et al., 2017). A mask video was created from the raw videos to detect the spots in each frame. To create the mask video, each frame was processed with background subtraction using the rolling‐ball method followed by filtering using Gaussian Blur and Median filters. Next, a threshold for each frame was set using the Bernsen local thresholding method and converted into a binary image. Detected spots in the mask video were filtered by area (> 40 nm^2^) and circularity (> 0.5). Frame‐to‐frame spot linking was performed using a Linear Assignment Problem tracker with a linking maximum distance of 20 nm, a gap‐closing maximum distance of 30 nm, and a gap‐closing maximum frame gap of 4. Only tracks with a minimum length of 30 frames were considered. Once the tracks were identified, the average spot intensity and area for each track was calculated and used to gate the tracks of interest.

### 2.10 Nano flow cytometry

The Nano flow cytometry analysis was performed using the Flow NanoAnalyzer (NanoFCM Co., LTD) according to the manufacturer's instructions. A Silica Nanospheres Cocktail (S16MExo, NanoFCM) was employed as the size standard to construct a calibration curve to allow the conversion of side scatter intensities to particle size. A concentration standard (200 nm PS QC beads, NanoFCM) was used to allow the calculation of particle concentrations. The laser used was a 488 nm laser at 25/40 mW, 10% ss decay. Detectors were equipped with 525/40 (AF488) and 580/40 (PE) bandpass filters. Antibodies were allowed to bind for 30 min on ice. Unbound antibodies were removed by centrifugation for 1 h at 100,000 g at 4°C. Labelled vesicles were then resuspended in 50 μL of PBS Buffer alone (PBS), unstained samples, and samples stained with isotype controls and auto‐thresholding were used to define sample and label specific signals.

### 2.11 Lipidomics

The extraction process began with taking approximately 250 μL of each of the two samples. Then, 200 μL of cold methanol followed by 800 μL of MTBE was added to each of these samples. The sample was mixed on a horizontal mixer for about 6 min. This was followed by addition of 200 μL of H_2_O, and centrifugation at 10,000xg for 10 min at 4°C. After centrifugation, the top layer was dried in a Speedvac for ∼2 h. The pellet was resuspended into 15 μL of Lipid resuspension solution, and 1 μL was loaded to MS/MS for high mass accuracy analysis. The intensities were normalized using the particle concentration based on NTA for the lipidomic analysis.

### 2.12 Proteomics

The EV samples (750 μL) were reduced with 5 μL of 10 mM tris(2‐carboxyethyl)phosphine and alkylated with 50 mM of iodoacetamide for 30 min each, acidified with 12% phosphoric acid 10:1 vol:vol, and then transferred to S‐trap columns. Samples were precipitated using 1:8 vol:vol dilution of each sample in 90% methanol in 100 mM Triethylamonium bicarbonate and digested with Trypsin (Promega, #V1115) overnight at 37° C. The samples were then run on a LC‐MS system comprised of an Evosep One and Bruker timsTOF Pro. For proteomic analyses, the raw files were searched against the reviewed Uniprot Homo sapiens database (retrieved. 2,01,80,131) using MaxQuant version 1.6.10.43. The intensities were normalized using particle concentration from NTA and further average‐based normalization was carried out to obtain the enriched set of proteins in each EV sample.

### 2.13 Total Internal Reflection Fluorescent Microscopy (TIRFM) and dSTORM imaging

Wheat Germ Agglutinin (WGA) conjugated with AlexaFluor488 (ThermoFisher Scientific; #W11261) and DiD/DiI (ThermoFisher Scientific; #V22887/#V22888) membrane dyes were used to label the EV samples captured on poly‐L lysine coated glass (Balint et al., 2020). Imaging was performed with an Olympus IX83 inverted microscope equipped with a TIRF module. The instrument was equipped with an Olympus UApON 150 × 1.45 NA oil‐immersion objective and Photomertrics Evolve delta EMCCD camera. Image analysis and visualization was performed using ImageJ software (Schneider et al., 2012) (National Institutes of Health). For dSTORM imaging, samples were mounted with a reducing buffer system and 10,000 images captured on a Nanoimager (Oxford Nanoimaging) with 100x oil‐objective lens and analyzed with Nanoimager Software v1.4.8740 (Oxford Nanoimaging).

### 2.14 Recombinant cell line generation

HEK293T cells were co‐transfected with lentiviral vector constructs, and packaging plasmids (PSPAX2 plasmid and pVSV‐G plasmid), and polyethyleneimine (PEI) mix for 48 h post‐transfection. Culture supernatants containing lentiviral particles were harvested and used to infect CD8^+^ T cells in media. Transduced cells were selected by addition of 2 μg/mL puromycin (Thermo Fisher Scientific) 48 h after transduction. Cells were maintained in selective media throughout.

### 2.15 Statistical analysis

Statistical analysis was performed using GraphPad Prism v9.3.1, and statistical significance between two groups was determined by unpaired t test (corrected by Holm‐Sidak method) after log_10_ transformation and normality test using Kolmogorov‐Smirnov test. Statistically significant differences are presented at probability levels of *p < 0.05, **p < 0.01, and ***p < 0.001.

## 3 RESULTS

### 3.1 Membrane spin filtration yields EVs with the highest level of impurities

Activated CD8^+^ T cells were used for the comparison of different: (1) EV release media (OptiMEM (Gibco, OptiMEM), RPMI‐1640 supplemented with 10% foetal bovine (exosome‐free) serum, and RPMI‐1640 without serum), (2) incubation time (24 h, 48 h, and 72 h), (3) starting culture densities (1 × 10^6^/mL, 2 × 10^6^/mL, and 5 × 10^6^/mL), and (4) EV isolation protocols (UC, MSF and SELC). We use analytical SELC and NTA size histograms, size versus intensity scatter plots and raw images to carry out an initial quality assessment with the goal of balancing yields of particles in range of sizes of EV with less soluble protein. As shown in Figure S1, although the EV containing peaks obtained for OptiMEM and RPMI‐1640 medias were similar, and smaller than R10, the contamination by particles with sizes < 80 nm is reduced in OptiMEM, and therefore this media was selected for subsequent studies. In OptiMEM, the 48 h time point yielded a greater number of > 80 nm particles than the 24 h time point, while extending to 72 h resulted in poor resolution of the EV containing peak by SELC (Figure S2). We chose 48 h as the standard duration for EV isolation. Although a cell density of 5 × 10^6^ activated cells/mL resulted in the highest EV peak, NTA revealed the presence of particles with sizes < 80 nm resulted in us choosing 2 × 10^6^/mL based on yield and initial purity (Figure S3). Thus, a cell density of 2 × 10^6^/mL activated CD8^+^ T cells in 15 mL of OptiMEM media for 48 h was used as input to compare the three isolation methods.

**FIGURE 1:**
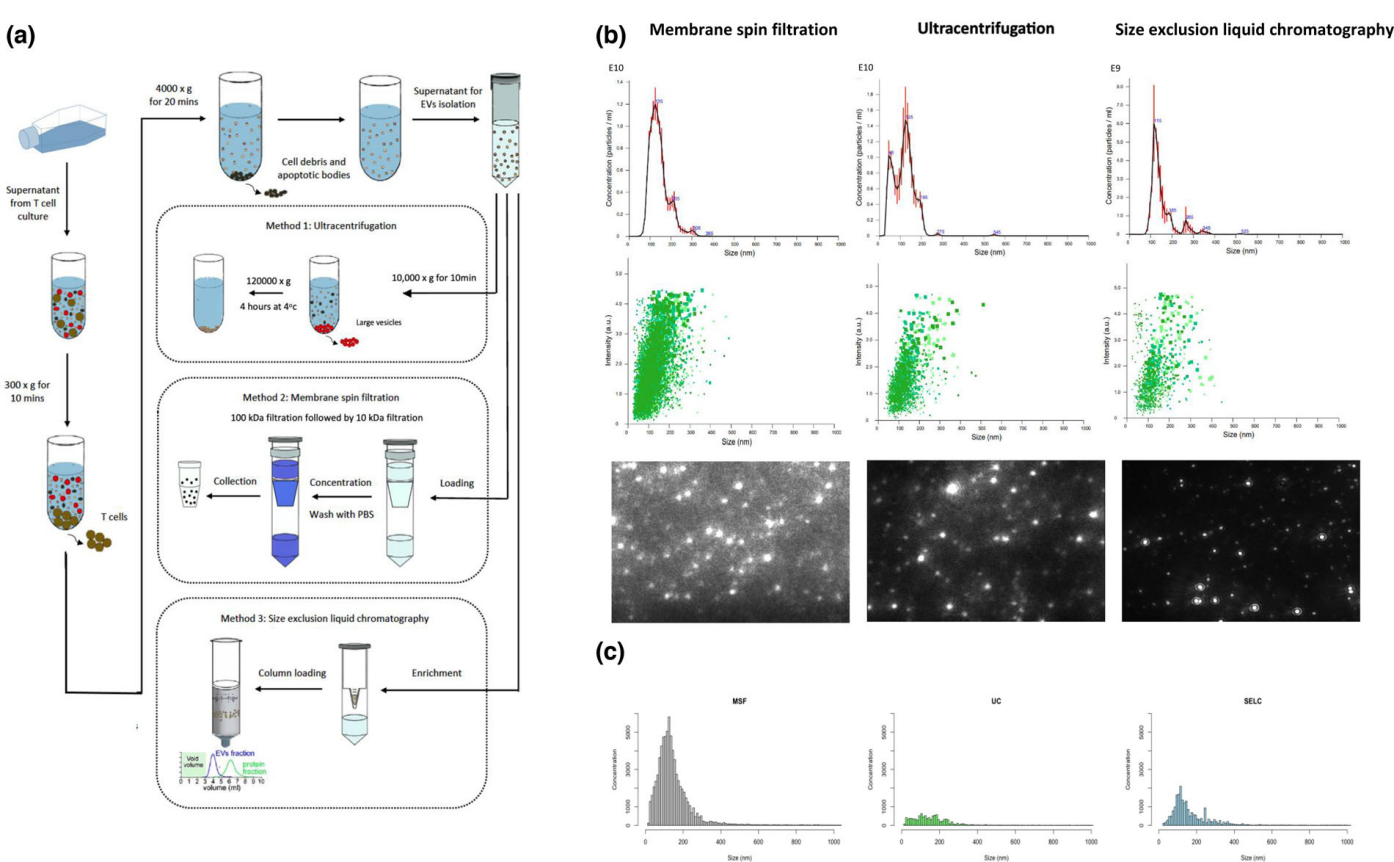
(a) Schematic showing the prominent methods that are commonly used for EV isolation. (b) Top panel: NTA analysis showing the concentration and size distribution of EVs obtained from different EV isolation protocols. Middle panel: size distribution intensities obtained for different isolation protocols. Bottom panel: Light scattering nanoparticle tracking image of the EVs isolated using different EV isolation protocols (*n* = 3). (c) Bar chart showing the size distribution versus concentration reflecting different isolation efficiencies with same Y axes scales (*n* = 3).

Figure 1(a) depicts the three EV isolation techniques evaluated. Cell culture supernatants were prepared by differential low speed centrifugations followed by UC, MSF or SELC. NTA was performed to check the concentration and size distribution of the particles isolated from the CD8^+^ T cells (Figure 1b, c and Figure S4). MSF and UC yielded higher concentrations of particles than SELC. However, SELC yielded particles enriched for target size range of EV (80–500 nm). Light scattering nanoparticle imaging of the EVs isolated using different EV isolation protocols (Figure 1b: bottom panel) reflects a similar observation.

**FIGURE 2:**
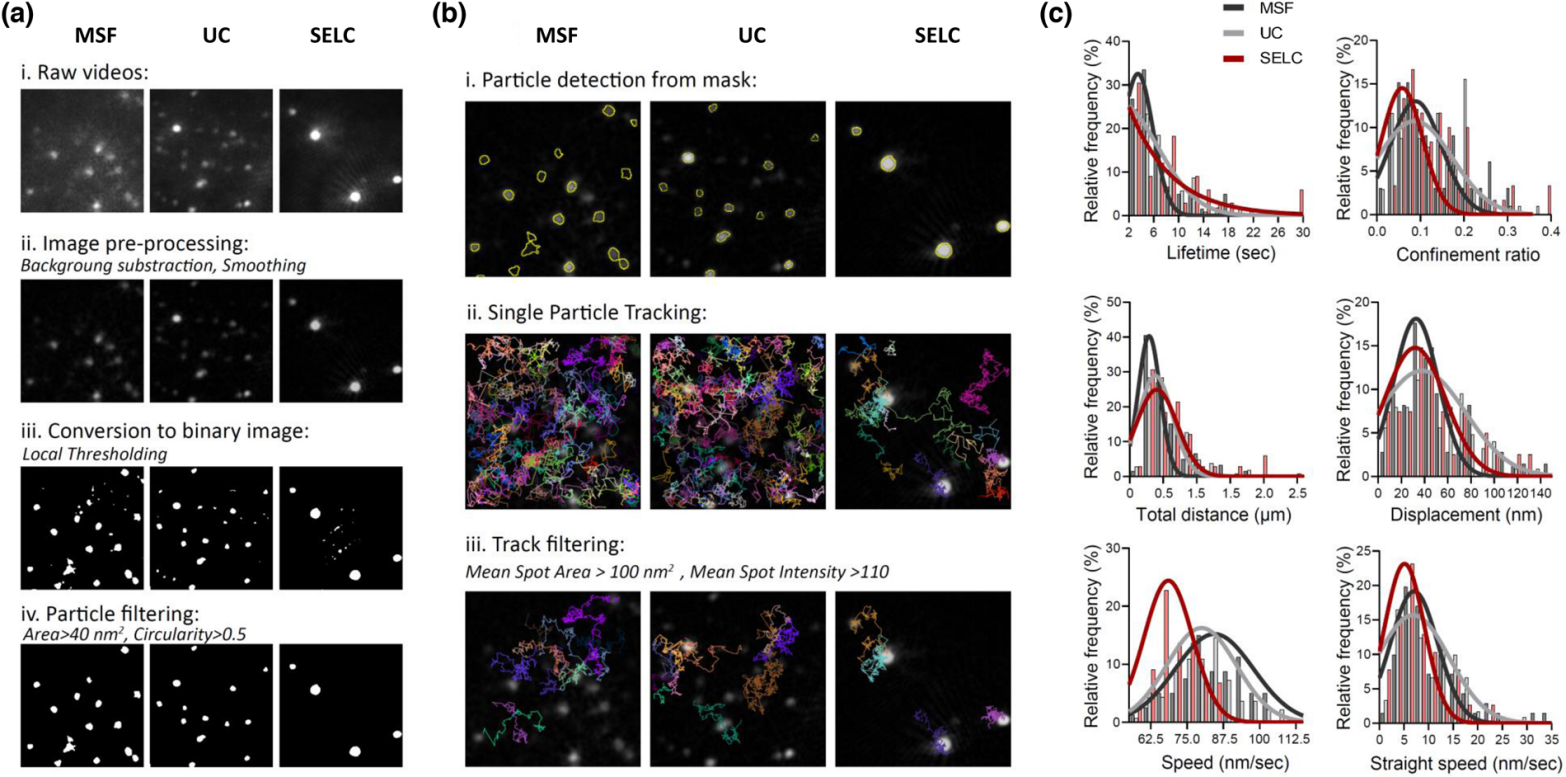
(a) Image processing workflow to convert the raw light scattering videos into a binary mask for particle detection: raw videos (i) are pre‐processed with background subtraction and smoothing filters (ii). Next, a threshold is applied to each frame allowing it to be converted into a binary image (iii) and detected particles are filtered based on size and circularity (iv). (b) Single‐particle tracking analysis workflow: particles in the videos are detected based on the generated mask videos (i) and tracks are estimated (ii). Tracks are then filtered based on the average spot area and intensity along the estimated track (iii). (c) Histograms showing the single particle tracking analysis data for each separation technique (Spin filtration, black; Ultracentrifugation, grey; Size‐exclusion liquid chromatography, red). Lifetime is the duration of the track. Displacement is the distance between the first and the last spot of the track in a straight line. Total distance is the full distance a particle travels throughout the track in nm, computed as the sum of all the link distances. Speed is the mean link velocity over all the links in the track. Straight speed is the mean straight line speed computed as the net displacement divided by the track duration. Confinement ratio is defined as the displacement divided by the total distance travelled by a particle (*n* = 3).

Single particle tracking analysis was performed to compare the nanoparticle concentration and size between isolation protocols. Light scattering videos were processed and particles in each frame were detected by converting the image into a binary mask. Particle frame‐to‐frame linking was performed and tracks were filtered based on the average intensity and area of the spots in the track. Track filtering enriched the sample sets with tracks containing bigger and brighter particles, reducing the particle heterogeneity between conditions. In addition, particle circularity increased after track filtering, indicating an improvement in the quality of the particles detected (Figure S8). Finally, tracks were analysed and kinetic parameters were compared between the different isolation protocols (Figure 2 and Figure S8). Duration of the tracks was higher for particles isolated by SELC, suggesting that the particles had a reduced mobility in the tridimensional space, which increased the time they remained in the focal space. In line with this observation, particles isolated by SELC showed a significant reduction in their speed compared to particles isolated by UC or MSF. In addition, the effective distance travelled during the duration of the track (displacement) was similar between conditions, even though particles isolated by SELC remained for longer in focus. Finally, the ratio between the particle net displacement and the total distance travelled (confinement) was lower when using SELC, indicating that these particles are less mobile in suspension.

**FIGURE 3:**
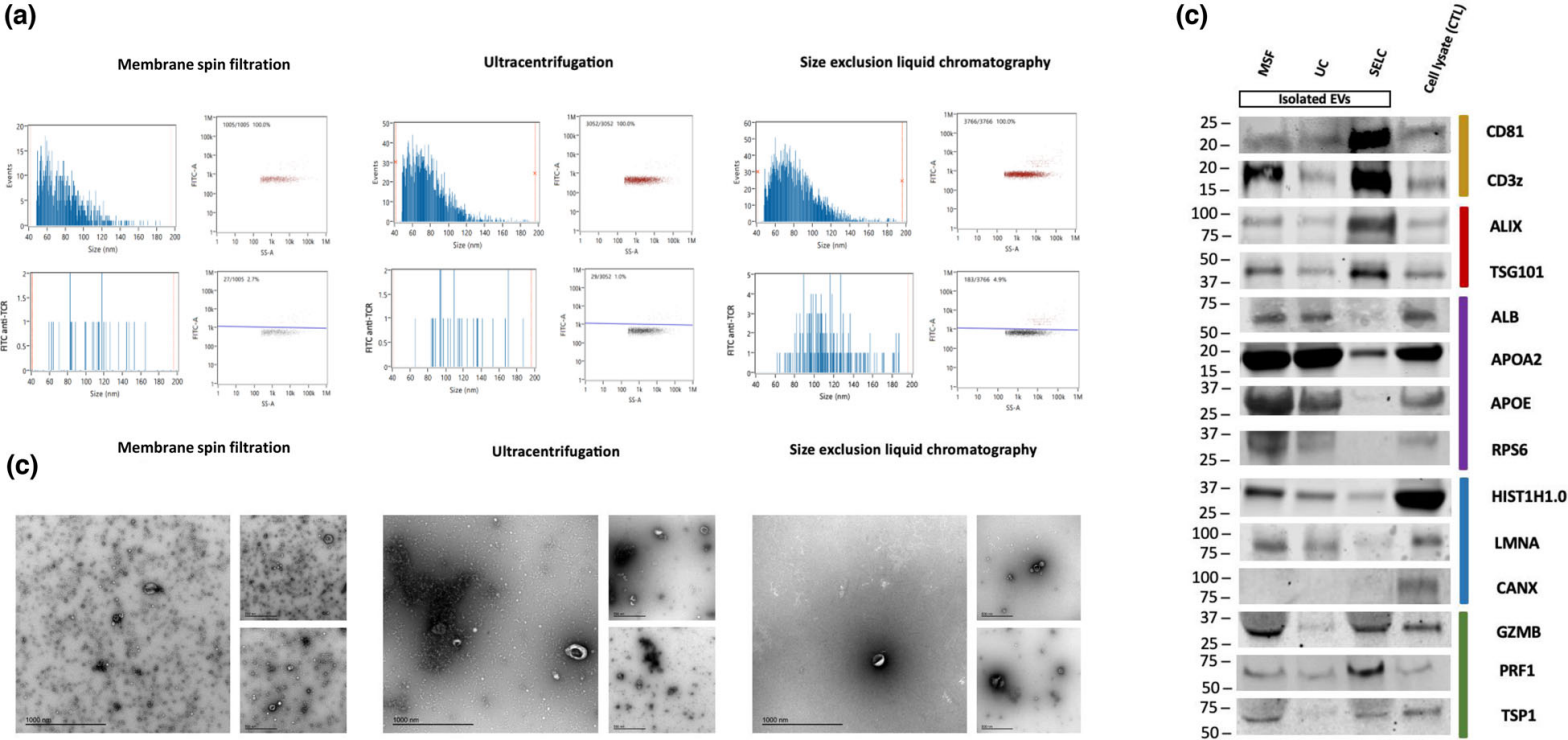
(a) NanoFCM analysis showing the scatter plot for TCR positive EVs marked in red above the blue line and the others below. Frequency of TCR positive events recorded on the stained EVs from different isolation protocols. (b) Transmitted electron microscopy images of eluates obtained using MSF, UC and SELC EV isolation technique at different scales (1000 nm and 500 nm) showcasing EVs and soluble proteins. (c) Western blot image for the lysed isolated EVs from different isolation protocols along with their cell lysate labelled for transmembrane proteins (yellow), cytosolic proteins (red), proteins that are major components of non‐EV co‐isolated structures (purple), transmembrane/lipid‐bound/soluble proteins associated with other intracellular compartments (blue), and secretory proteins (green).

To further investigate the difference in the yield and purity of T cell particles isolated using MSF, UC and SELC, we performed nano flow cytometry (NanoFCM) and electron microscopy studies (Figure 3a and b, respectively). NanoFCM confirmed that the particle size distribution followed the trend SELC > UC > MSF (Figure 3a). The T cell antigen receptor is expected to be present on the surface of T cell‐derived EVs (Hivroz et al., 2017). The percentage of TCR^+^ vesicles was higher when T cell‐derived particles were isolated by SELC (4.9%), followed by MSF (2.7%), and lastly by UC (1.0%) (Figure 3a). Transmission electron microscopy of samples isolated by the different methods revealed the presence of EVs with the typical round and cup shaped morphology in all samples, but also the presence of smaller non‐vesicular particles mainly in samples isolated by MSF and UC. In contrast, samples obtained from SELC showed the lowest degree of contaminating non‐vesicular particles (Figure 3b). In order to test the efficiency of isolation techniques, we also transduced EGFP tagged CD63 reporter in CD8^+^ T cells and performed NanoFCM analysis on the purified extracellular particles. The T cell derived particles that were isolated using SELC were seen to have the highest percentage of positive events (38.4%) (Figure S5).

Immuno‐blotting analysis of the particle fractions and cell lysates from activated CD8^+^ T cells using MSF, UC, and SELC was performed (Figure 3c). Samples were probed with antibodies to detect (Uhl & Gérard, 2020) transmembrane proteins typically enriched on EVs (CD81, a non‐tissue specific EV marker and CD3z, a T cell specific EV marker), (Basu & Huse, 2017) cytosolic endosomal sorting complex required for transport (ESCRT) components, widely used as EV markers (PDCD6IP (ALIX) and TSG101), (Sowinski et al., 2008) non‐EV co‐isolated proteins (ALB and APOA2), (Uhl & Gerard, 2020) transmembrane/lipid‐bound/ soluble contaminant proteins, associated with intracellular compartments (HIST1H1.0, LMNA and CANX), and (Balint et al., 2020) T cell‐derived secretory proteins associated with SMAPs (TSP1, PRF1 and GZMB). Transmembrane and cytosolic EV marker proteins were enriched in SELC‐purified particles when compared to particles isolated using MSF or UC. Most non‐EV marker proteins (ALB, APOA2, HIST1H1.0, LMNA, and CANX) were relatively more depleted in particles isolated by SELC when compared to MSF and UC purified particles. SMAP components were enriched in particles isolate by MSF and SELC and seemed depleted in UC, suggesting that SMAPs may not be recovered well from UC pellets. Since the particles isolated from T cells using MSF had higher levels of contaminants present in the purified sample, further analyses were carried out using particles enriched by UC and SELC.

We also performed iodixanol gradient and evaluated its efficacy in purifying CD8^+^ T‐cell EVs to check if the ribosomal proteins are less in the EVs purified using UC (Figure S6). Electron microscopy and western blotting were performed to validate the EV fraction. However, the resultant concentrations of EVs isolated were low. The NanoFCM measurements for the TCR^+^ EVs were further quite low in comparison to the other technologies evaluated for the same initial conditions of supernatant from the same given donor. In order to improve the quality of particles isolated using UC, we also tried washing the pellet and performing re‐UC; however, this considerably reduces the yield of EVs and TCR^+^ EVs (Figure S7). Thus, none of the downstream characterizations were performed due to the limiting concentration of the EVs isolated using both of the above techniques.

### 3.2 SELC enriched EVs and SMAPs

**FIGURE 4:**
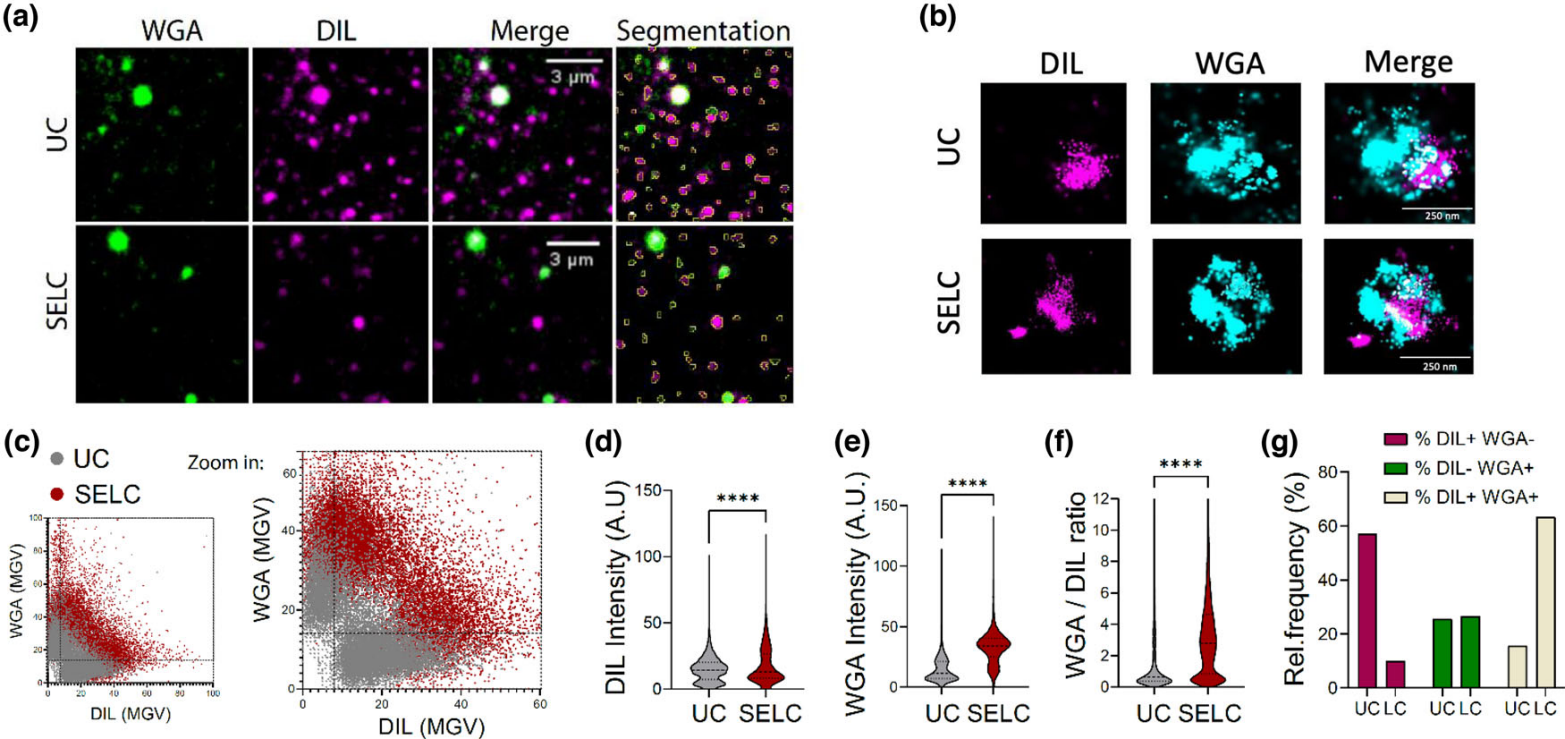
(a) Purified EVs were stained for glycoproteins (WGA, green) and membranes (DIL, magenta) and imaged with TIRFM. WGA positive and/or DIL positive particles were segmented and mean intensity grey values (MGV) were analysed within the particle for both WGA and DIL. (b) Representative two‐colour dSTORM imaging of EVs samples purified using UC or SELC. EVs were stained with WGA and DIL. (c) Scatter plot showing the intensity for both WGA and DIL per each detected particle, for both UC (grey) and LC (red). Bottom plot shows the zoomed in area for the purpose of increased resolution. (d‐f) Violin plots of the distribution of the DIL intensity (lipids), WGA intensity (glycoconjugates) and the WGA/DIL intensity ratio for the particles detected in the TIRFM images, UC (grey) or SELC (red). Data shows the frequency distribution of > 14,000 particles. Lines show the median and quartiles. ****pv < 0.001, Mann Whitney test. (g) Single and double positive populations for both DIL and WGA were quantified using the gates applied in C. Data is shown as the % population from the total particles detected for both UC and LC samples (*n* = 3).

We next assessed the macromolecular composition of the particles obtained with UC or SELC. Particles were captured on poly‐L‐lysine coated glass, stained fluorescently tagged wheat germ agglutinin (WGA), a lectin that detects glycoproteins, and DIL, a lipophilic fluorescent dye, and imaged using TIRFM (Figure 4a). dSTORM imaging confirmed that the WGA dominant and DIL dominant staining particles were distinct, although they were sometimes adjacent to each other on the surface as if they may interact (Figure 4b). EVs are expected to be relatively more DIL positive, whereas glycoprotein‐based particles like SMAPs are expected to be more WGA positive. The overall staining intensity in SELC enriched particles was higher for both WGA and DIL compared to the UC enriched particles (Figure 4c–g). The reason for this was not clear as the input material was the same. However, if this intensity different is set aside, the UC and SELC preparations each have a population of WGA bright and DIL bright particles which may correspond to SMAPs and EVs, respectively. The SELC particles are 90% WGA bright, whereas the UC particles are 40% WGA bright (Figure 4g). These results are consistent with greater preservation of SMAPs by SELC compared to UC.

**FIGURE 5:**
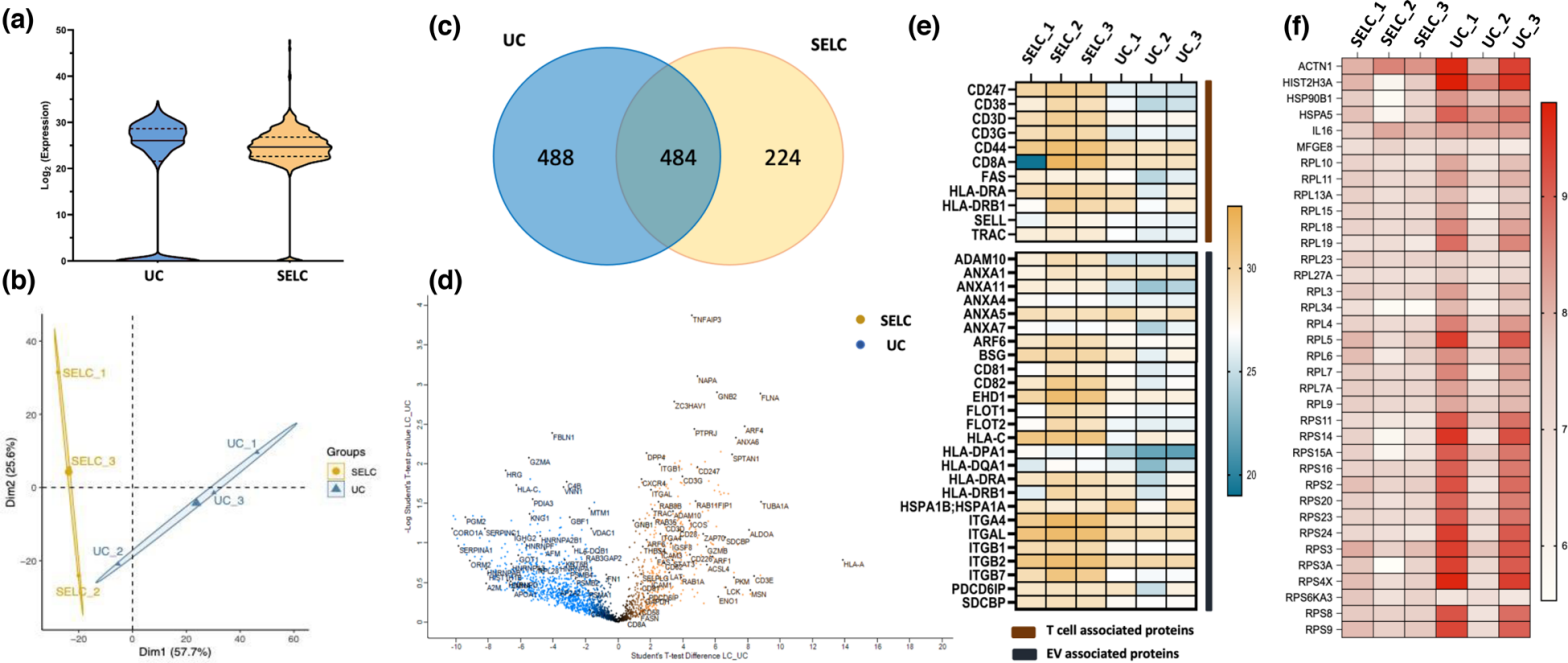
(a) Violin plot showing the distribution of the protein contents in the EVs isolated using SELC (yellow) and UC (blue). (b) PCA biplot of the samples showing variation in the protein profiles between the EVs isolated using SELC (yellow) an UC (blue). (c) Venn diagram depicting the numbers of shared and unique enriched proteins between the two samples. (d) Volcano plot representing the differentially expressed proteins in EV samples isolated using SELC (yellow) and UC (blue). (e) Heatmap showing the expression of T cell‐specific markers and EV markers across three biological replicates of the EV samples isolated using SELC and UC. The intensity values were log2 transformed. (f) Heatmap showing the expression of non‐EV markers (contaminating proteins) across three biological replicates of the EV samples isolated using SELC and UC. The intensity values were log10 normalised (*n* = 3).

Proteomic analysis showed the distribution of abundances for the 972 and 708 proteins detected in particles isolated by SELC and UC, respectively (Figure 5a) (Supplementary data 1). Particles prepared by SELC were more homogeneous than those isolated by UC based on the percentage of proteins around the median expression being higher. In addition, SELC particles had fewer proteins with very low expression levels when compared to UC particles. The principal component analysis biplot (Figure 5b) shows that particles isolated using SELC or UC were sufficiently distinct and were well separated by dimension 1, but showed significant variation between donors in a second dimension.

The number of the enriched proteins that are common and unique to the particles isolated using the different methods were depicted using a Venn diagram (Figure 5c), where 488 unique proteins were found to be enriched in particles isolated by UC and 224 in particles isolated by SELC. The volcano plot (Figure 5d) highlights the differentially enriched proteins in the particles isolated using SELC or UC. ADAM10, ICAM1, TRAC, and ZAP70 were found to be highly enriched in particles isolated using SELC, whereas HIST1H1B, PGM2, SERPINA1 were enriched in particles isolated using UC.

We next assessed the quantities of T cell surface proteins and EV associated proteins (based on MISEV guidelines) (Théry et al., 2018) in particles isolated with each method, in three independent experiments. T cell surface proteins and EV‐associated proteins were found to be enriched in particles isolated by SELC, when compared to particles purified by UC (Figure 5e). Proteins that were enriched in SELC particles included the transmembrane metalloprotease ADAM10, the adenosine generating ectoenzyme CD38, and the tetraspanin CD81. The expression of non‐EV markers (based on MISEV guidelines) (Théry et al., 2018) (Figure 5f) including secretory proteins (IL16, MFGE8), transmembrane/ lipid‐bound/ soluble proteins associated with intracellular compartments other than endosome or plasma membrane (HSPA5, ACTN1) and proteins that are major components of non‐EV co‐isolated structures (e.g., ribosomal proteins such as RPL11, RPS11), were found to be more expressed in particles isolated by UC, when compared to SELC purified particles. This clearly shows that particles isolated employing SELC result in the lowest level of contaminants.

### 3.3 The purification method selects for different origins of particles

**FIGURE 6:**
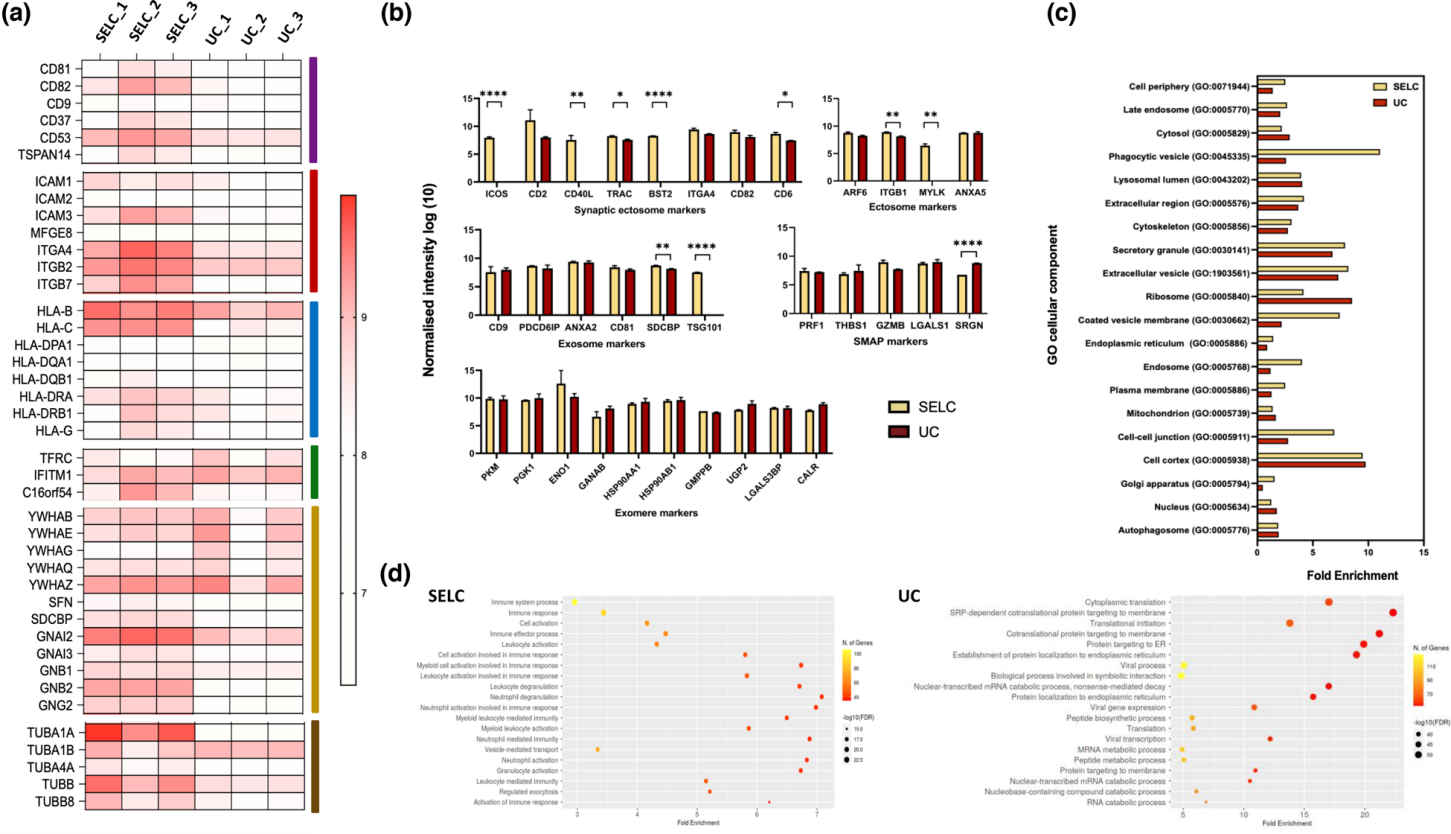
(a) Heatmap depicting the expression of different major protein classes across three biological replicates of the EV samples isolated using SELC and UC. The major proteins of different protein classes, namely, tetraspanins (purple), adhesion molecules (AMs) (red), antigen‐presenting molecules (APMs) (blue), transmembrane proteins (TMPs) (green), signal transduction proteins (STPs) (golden), and cytoskeletal proteins (CPs) (brown). The intensity values were log10 transformed. (b) Bar charts showing the expression of different EV class markers: Synaptic ectosome, Ectosome, Exosome, SMAPs, and Exomere across the EV samples isolated using SELC (yellow) and UC (red). The intensity were log10 normalised. Statistical analysis was performed using GraphPad Prism v9.3.1, and statistical significance between two groups was determined by unpaired t test (corrected by Holm‐Sidak method) after log10 transformation and normality test using Kolmogorov‐Smirnov test. Statistically significant differences are presented at probability levels of *p < 0.05, **p < 0.01, and ***p < 0.001. (c) Bar chart showing the fold enrichment—Gene Ontology (GO) cellular component analysis of the enriched proteins found in the EVs isolated using SELC (yellow) and UC (red). (d) Bubble plot depicting the fold enrichment of GO molecular function of the enriched proteins found in the isolated EVs. The size of the bubble represents the –log10(FDR) and the colour scale represents the number of genes (*n* = 3).

To further investigate the EV and SMAP protein composition and determine whether different isolation methods result in EVs from distinct origins, the expression levels of various protein and EV marker classes were analysed. Figure 6(a) depicts the relative abundance of different protein classes (Andreu & Yáñez‐Mó, 2014; Colombo et al., 2014; Jurj et al., 2020; Jankovičová et al., 2020; Willms et al., 2018) quantified in particles isolated by SELC or UC, namely, tetraspanins (purple), adhesion molecules (AMs) (red), antigen‐presenting molecules (APMs) (blue), transmembrane proteins (TMPs) (green), signal transduction proteins (STPs) (golden), and cytoskeletal proteins (CPs) (brown). A higher abundance of tetraspanins (CD81, CD82, CD53) and AMs (ITGA4, ITGB7) was observed in particles isolated using SELC compared to UC. Similarly, the abundance of most APMs (HLA‐C, HLA‐DRA), TPMs (TFRC), STPs (GBM2, SDCBP), and CPs (TUBA1A, TUBB8) were also comparatively higher in the particles isolated using SELC. However, abundance of certain tetraspanins (CD9), AMs (MFGE8), APMs (HLA‐DPA1, HLA‐DQA1) did not show variation with the different isolation technique.

Figure 6(b) shows the abundance of different classes of EV and SMAP markers across the particles isolated by SELC or UC. Most synaptic ectosome markers (Saliba et al., 2019) had a higher expression level in the particles isolated using SELC when compared to UC. The difference in the abundance of markers belonging to this class were statistically significant in proteins such as costimulator ICOS, membrane anchored CD40L (*p* ≤ 0.01), BST2 (*p* ≤ 0.0001), CD6 (*p* ≤ 0.0001) and TRAC (*p* ≤ 0.05). The expression levels of ectosome/microvesicle markers (Kalra et al., 2016, Muralidharan‐Chari et al., 2009) MYLK (*p* ≤ 0.01), a regulator of cytoskeletal contractility, and ITGB1 (*p* ≤ 0.01), the integrin adhesion molecules subunit were also higher in SELC purified particles, when compared to UC purified particles, whereas the small G protein ARF6, and the phosphatidylserine binding protein ANXA5 were less sensitive to the isolation method. The expression levels of exosome markers (Jaiswal & Sedger, 2019; Kim et al., 2017; Keerthikumar et al., 2016; Zhang et al., 2018), such as syntenin‐1 SDCBP (*p* ≤ 0.01) and the ESCRT‐I complex component TSG101 (*p* ≤ 0.0001), were significantly lower in particles isolated using UC than SELC. In contrast, the expression levels of the tetraspanins CD9 and CD81, the ESCRT component PDCD6IP (also referred to as ALIX), and ANXA2 did not vary. The SMAP marker (Balint et al., 2020; Chang et al., 2022) SRGN (*p* ≤ 0.0001) had a significantly higher expression level in the particles isolated using UC than SELC, but as its uncorrelated with other SMAP components this difference may reflect involvement in other types of protein particle released from CD8^+^ T cells. Exomere markers (Sheehan & D'Souza‐Schorey, 2019; Zhang et al., 2018) such as CALR, GANAB, PGK1, ENO1 and PKM did not show much variation in the expression levels with the different isolation methods.

To examine the origin of the enriched proteins found in these vesicles, Gene Ontology (GO) cellular component analysis was performed (Figure 6c). The score for GO terms including ‘Extracellular vesicle’, ‘Phagocytotic vesicle’, ‘Late endosome’, ‘Endosome’, ‘Plasma membrane’, ‘Coated vesicle membrane’, and ‘Cell‐cell junction’ were comparatively higher in the particles isolated using SELC compared to UC. In contrast, GO terms such as ‘Ribosome’, and ‘Cytosol’, were found to have a comparatively higher score in the particles isolated using UC (Figure S9). As the functionality of particles depends greatly on their composition, GO molecular function on the enriched proteins was performed. The results are shown in Figure 6(d) as a bubble plot, where the number of genes associated with each GO functional category, their fold enrichment and –log10(FDR) value are presented for each function. The particles isolated by UC had a higher fold enrichment in GO terms such as ‘Cytoplasmic translation’, ‘Protein targeting to ER’ and ‘Cotranslational protein targeting to membrane’. In contrast, GO terms such as ‘Neutrophil degranulation’, ‘Leukocyte degranulation’, and ‘Granulocyte activation’, related to immune response were found to be enriched in the particles isolated using SELC. In conclusion, the proteomic profiles of the particles isolated using SELC and UC showed distinct variations with greater enrichment for EV and SMAP signatures in SELC than UC overall, even though the particles were obtained from the same cell of origin and following the same culturing conditions.

### 3.4 Lipid content in particles is dependent on the isolation technique

**FIGURE 7:**
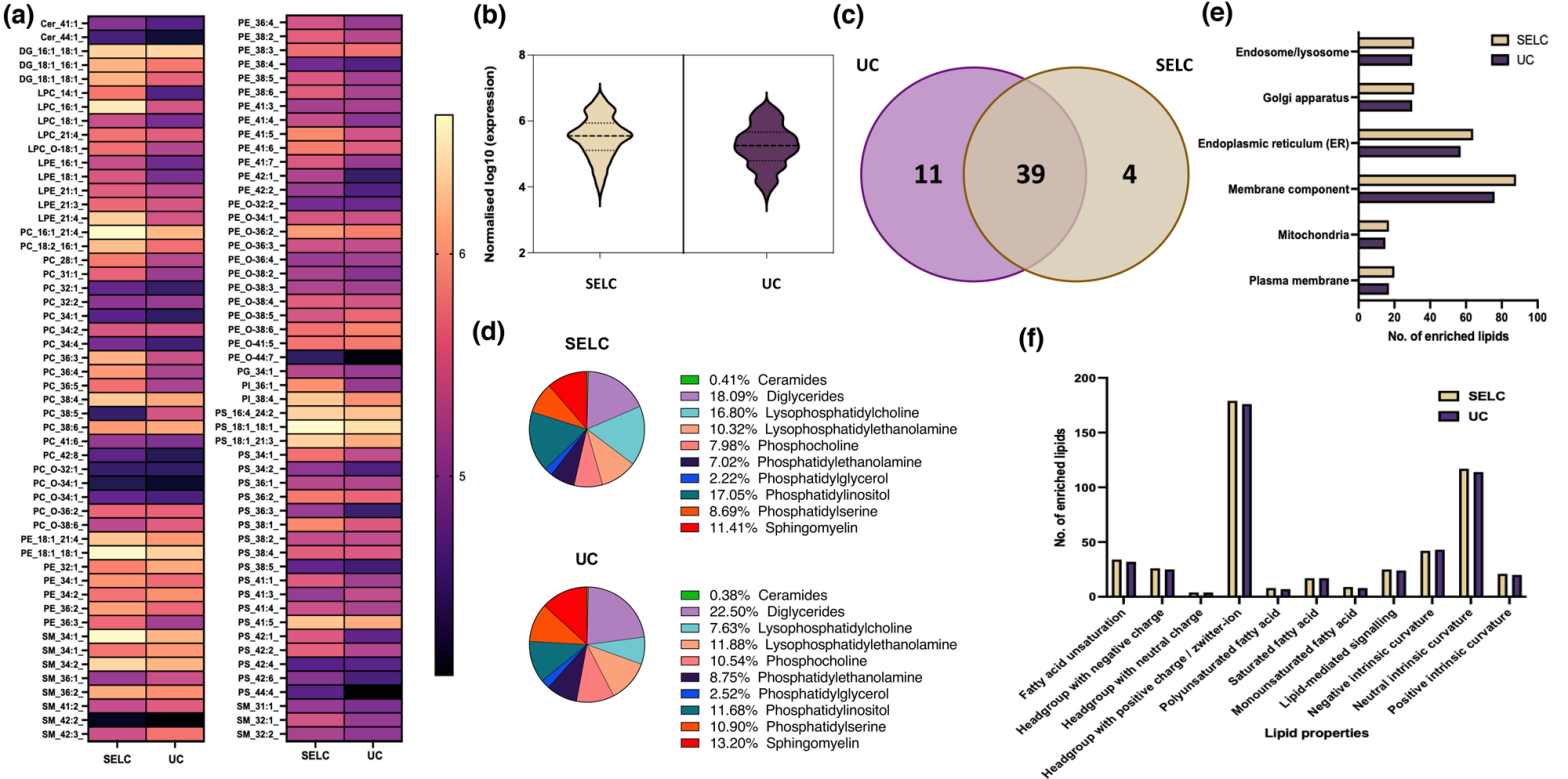
(a) Heatmap depicting the expression of lipids from the EV samples isolated using SELC and UC. The intensity values were log10 transformed. (b) Violin plot showing the distribution of the lipid contents in the EVs isolated using SELC (yellow) and UC (purple). (c) Venn diagram depicting the numbers of shared and unique enriched lipids between the two samples. (d) Pie chart depicting lipid class distribution in EVs isolated using SELC and UC. (e) Bar chart showing the number of enriched lipids—Gene Ontology (GO) cellular component analysis of the enriched lipids found in the EVs isolated using SELC (yellow) and UC (purple). (f) Bar chart showing the number of enriched lipids under different lipid property categories found in the EVs isolated using SELC (yellow) and UC (purple).

Lipidomic analysis was performed to further investigate the lipid composition, membrane biogenesis and functionality of the particles obtained using SELC or UC (Supplementary data 2). Figure 7(a) depicts the expression level of each lipid in the particles as a heatmap. Classes of lipids such as lysophosphatidylcholines (LPC), phosphatidylcholines (PC), and ceramides (Cer) were more abundant in the particles isolated by SELC. In general, the lipid classes diglycerides (DG) and phosphatidylinositol (PI) were found to be highly abundant in EVs irrespective of the isolation technique. Figure 7(b) shows the distribution of the expression (log10) of lipid contents in the particles isolated using SELC and UC. The median expression level of the lipid was comparatively higher in T cell particles obtained by SELC than UC. However, the number of lipid species around the median expression level was higher in particles obtained using UC than SELC, suggesting that UC isolates a broader range of lipid species, of which sphingomyelins stood up over other lipid species. This observation is consistent to the increased recovery of lipoprotein components in UC proteomics (Figure 3c and Figure S9B) (Rye et al., 1996).

Figure 7(c) shows the number of the enriched lipids common and unique to the particles isolated using SELC and UC, 39 enriched lipids were common, and 11 and 4 enriched lipids were unique to particles isolated using UC and SELC, respectively. The lipid class distribution in particles isolated using SELC and UC is depicted as the pie chart in Figure 7(d). It was seen that the highly abundant lipid classes in particles isolated using SELC were diglycerides (18.09%) followed by phosphatidylinositol (17.05%), and similarly, the highest abundant lipid class in particles isolated using UC were diglycerides (22.50%), but followed by sphingomyelin (13.20%).

Following this, we investigated the subcellular origin of the lipid and the lipid physicochemical properties depicted as bar charts in Figure 7(e) and (f), respectively. Lipids from the ‘Endoplasmic reticulum (ER)’, ‘Membrane component’, ‘Endosome/lysosome’ and ‘Golgi apparatus’ were found to be highly abundant in isolated particles irrespective of the isolation protocol employed (Figure 7e). However, these lipids were relatively more abundant in particles obtained using SELC than UC. Figure 7(f) shows that lipids with ‘Headgroup with positive charge/ zwitterion’ and ‘Neutral intrinsic curvature’ were found to be highly abundant, whereas the lipids with ‘Headgroup with neutral charge’ were found to be the least abundant lipid category in the particles isolated using SELC and UC. The number of enriched lipids under most categories based on their property was slightly higher in the EVs isolated using SELC than UC. Thus, our lipidomic results provide an additional piece of evidence for the impact of different EV isolation techniques on the biomolecular profiles identified.

## 4 DISCUSSION

This study evaluates the purity and composition of particles isolated from CD8^+^ T cells using three conventional isolation techniques–UC, MSF and SELC. The isolation conditions were standardized, and equivalent parameters were set for all the techniques to minimize any interference in the results. Similarly, other reports also have shown isolation techniques to influence the outcome of the purity and concentration of the extracellular particles isolated from plasma, body fluids, and various cell types (Brennan et al., 2020; Dong et al., 2020; Mol et al., 2017; Ter‐Ovanesyan et al., 2021; Takov et al., 2019). Mol et al. (2017), compared UC and size exclusion liquid chromatography for isolating EVs from cardiomyocytes progenitor cells (CPC) and showed that EVs purified by size exclusion chromatography harbour better biophysical functionalities (Mol et al., 2017). Dong et al 2021, also compared UC and size exclusion chromatography for isolating EVs from serum, cell culture medium and urine samples. Their findings show that irrespective of sample types, size exclusion chromatography resulted in isolating pure EV fractions (Dong et al., 2020). Herein, we have investigated potential EV isolation methods for purifying extracellular particles from human CD8^+^ T cells. The NTA analysis showed that the concentration of the particles obtained was higher in the case of MSF followed by UC, with the lowest concentrations obtained when EVs were isolated by SELC. However, electron microscopy images reflect that the sample contaminants were higher in MSF and UC compared to SELC. Furthermore, NTA analysis demonstrates that isolation of EVs by SELC yields distinct EV populations with a range of expected size. Single particle tracking analysis shows distinct kinetics for the particles isolated using SELC, which show a reduced motion in the suspension media, when compared by particles isolated using MSF or UC. Reduced mobility of the SELC particles in suspension compared to the UC or MESF could be due to larger size and/or roughness (Cai et al., 2018; Dehghani et al., 2020; Guazzelli & Morris, 2011). Subsequently, the western blotting was performed on the particles samples following the ISEV guidelines (Théry et al., 2018), where a minimum of two proteins was considered for each category‐ (i) transmembrane or GPI‐anchored proteins associated to plasma membrane and/or endosomes, (ii) cytosolic proteins recovered in SMAPs, (iii) major components of non‐EV co‐isolated structures and (iv) transmembrane, lipid‐bound and soluble proteins associated to other intracellular compartments than PM/endosomes, which can be grouped under EV markers and non‐EV markers. Immunoblotting results confirmed that non‐EV markers were present in high or moderate levels in particles obtained using UC and MSF, while EV markers were found to have higher expression levels in particles isolated using SELC. We also performed flow nanoparticle analysis using the flow nanoanalyser from NanoFCM and observed that the concentration and the percentage of TCR^+^ EVs were higher in the particles isolated using SELC, which suggest that isolation technologies variably influence the purity of EVs. Importantly, SELC provides an alternative method to BSLBs to obtain highly pure and concentrated preparations of TCR^+^ EVs, which are constitutively secreted in scarce amounts by activated T cells (Céspedes et al., 2022). The latter is key when studying the composition and biogenesis of TCR^+^ EVs to develop cellular platforms for their recombinant production. Due to the high levels of contamination found when isolating particles with MSF, we did not continue using this technique in subsequent experiments.

Previous studies looking at EVs released by primary and tumour T cells (Jurkat) predominantly focus on using UC for EV isolation and functional activation of antigen‐presenting cells (Fu et al., 2019; Seo et al., 2018; Tung et al., 2018, 2020). These include studies showing that EVs contain TCR/CD3 on their surface and that EVs can trigger anti‐viral responses in dendritic cells (DC) (Blanchard et al., 2002) or antibody production in B cells (Carpier et al., 2018) through the transfer of nucleic acids. Other studies using affinity purification of Strep‐tagged transmembrane proteins showed the function of LAT‐containing membranes on T cell activation (Hivroz et al., 2017). EVs released by NK cells and CTLs are also emerging as anticancer autonomous entities with therapeutic potential (Del Vecchio et al., 2021). EVs purified from cytotoxic T cells using UC have proved efficient for the control of tumour progression. For example, mesenchymal tumour stromal cells engulf RNAs contained in cytotoxic EVs, which correlates with the depletion of these cells (Seo et al., 2018). UC was also used to purify exosomes from CAR‐T cells, which contained the CAR on their surface and were enriched with cytotoxic molecules that inhibited tumour growth. In addition, CAR‐T cell exosomes did not contain PD1 and thus, their anti‐tumour activity was not reduced by PD‐L1, overcoming the sensitivity of parental CAR‐T cells to immunosuppressive tumour microenvironment (Fu et al., 2019). Combination of UC with immune‐isolation was also used to separate distinct types of fusion‐competent granules from cytotoxic T cells, which include, but are not limited, to SMAPs. These different studies have functionally showed the potential of T cell‐derived EVs in inducing immunomodulatory and cytotoxic activity. Here, we extend our understanding on the technical procedures required for the optimal isolation of constitutively released EVs using several techniques, including proteomics and lipidomics.

T cells' particles obtained using UC and SELC were further evaluated based on their protein and lipid composition. The abundance level of the proteins in the particles varied depending on the isolation protocol employed. SELC particles tend to be more proteinaceous, while their relative amount of lipids remained similar to particles isolated with UC. The identity of the proteins enriched in the particles was also dependent on the isolation method. Most of the T cell‐associated and EV‐associated proteins were found to be enriched when particles were obtained using SELC. The non‐EV marker proteins had higher expression in the particles obtained using UC, which was evident from the volcano plot and the heatmap. From the expressions of EV classification markers, it was apparent that the particles isolated using SELC had a higher number of proteins associated with ectosomes and synaptic ectosomes than those obtained using UC. However, the differential abundance of exomeres, exosomes and SMAPs in the particles purified using these techniques was not distinct, as there was no clear trend in the expression level of the markers. In addition, tetraspanins, AMs, and APMs were comparatively abundant in the particles obtained using the SELC isolation protocol. Besides, there was no evident difference observed for STPs, CPs and TMPs, and thus, it is not feasible to comment on their abundance in the particles obtained.

Based on the GO cellular component analysis, the proteins from particles obtained using SELC were most likely be expected to be originated from the phagocytic vesicle, endosomes, plasma membrane, secretory granules and cell‐cell junction; in contrast, those obtained using UC most likely originated from the cell cortex, cytosol and the ribosome. Besides, it can also be observed that proteins related to T cell or immune functions were highly enriched in the particles isolated using SELC, whereas the proteins related to metabolic, catabolic, and translation processes were highly abundant in the particles obtained using UC. This clearly shows that UC as protocol for isolating extracellular particles from CD8^+^T cells copurifies a lot of impurities which may hampers downstream applications for therapeutic purposes.

Lipid components has been shown to influence the functionality of CD8^+^ T cells through exposing phosphatidylserine and thereby sequestering and inactivating perforin. Thereby it was essential to study the lipid composition of the particles released by these T cells. Our lipidomics study (Venn diagram and heatmap) suggests that fewer lipids were present in very high abundance in the particles isolated using SELC than UC. Besides, the lipid composition and lipid nature varied depending upon the isolation protocol employed. For example, a few lipid classes like Diglycerides and Phosphatidylinositol constituted a higher proportion of the whole lipid content in the particles obtained using SELC comparatively to UC where the highest abundant lipid class in EVs were also diglycerides but followed by sphingomyelin instead of phosphatidylinositol. Importantly, it has been reported that phosphoinositides, including PIP_2_ along with diglycerides, are crucial regulators of T cell activation, migration and immunological synapse formation by majorly regulating the actin cytoskeleton and downstream signalling cascade (Porciello et al., 2016). Also, given that sphingomyelins are the most abundant sphingolipids in lipoproteins (Iqbal et al., 2017), the low abundance of sphingomyelins in SELC compared to UC suggests a better exclusion of lipoproteins in SELC than UC (Martinez‐Beamonte et al., 2013; Simonsen, 2017).

## 5 CONCLUSION

Overall, it was evident that for the same cell source of origin and keeping the culturing conditions constant, the isolation protocol employed had an impact on the EV protein and lipid content as well on the concentration and purity of the EVs obtained. Furthermore, we observed a trade‐off between the concentration and the purity of the particles obtained. Our studies provide further evidence that, depending on the intended purpose of the EVs, either for functional studies, characterization, or engineering, the selected isolation protocol might interfere with the results obtained. This work serves as a template for comparative analysis of purifying extracellular particles from different T cells subtypes. This work has shed light on specific techniques like MSF that should be avoided when isolating EVs and SMAPs from CD8^+^ T cells, and most likely from any other cell type as well. UC was seen to be better at enriching extracellular particles derived from intracellular compartments. Our study found SELC to be the most appropriate methodology of the tested techniques for purifying mixtures of EVs and SMAPs from CD8^+^ T cells isolated from human PBMCs, resulting in higher purity of EVs with co‐purification of SMAPs. The EV component had protein and lipid compositions that better represent well know EV markers and the composition of the cell of origin.

## AUTHOR CONTRIBUTIONS

**Ashwin K. Jainarayanan**: Conceptualization; Data curation; Formal analysis; Investigation; Methodology; Validation; Visualization; Writing‐original draft; Writing‐review & editing. **Jesusa Capera**: Formal analysis; Investigation; Visualization; Writing‐review & editing. **Pablo F. Céspedes**: Investigation; Supervision; Validation; Writing‐review & editing. **Mariana Conceição**: Conceptualization; Supervision; Validation; Writing‐review & editing. **Mirudula Elanchezhian**: Formal analysis; Investigation; Visualization; Writing‐original draft. **Tom Thomas**: Formal analysis; Investigation; Visualization; Writing‐review & editing. **Scott Bonner**: Formal analysis; Validation; Writing–review & editing. **Salvatore Valvo**: Data curation; Visualization; Writing‐review & editing. **Elke Kurz**: Project administration; Resources; Validation; Writing‐review & editing. **Ranjeet Singh Mahla**: Investigation; Validation; Visualization; Writing‐original draft. **Georgina Berridge**: Data curation; Formal analysis; Methodology; Resources. **Svenja Hester**: Data curation; Formal analysis; Methodology. **Roman Fischer**: Data curation; Investigation; Methodology; Resources. **Lynn B. Dustin**: Investigation; Resources; Supervision; Writing‐review & editing. **Matthew J. A. Wood**: Conceptualization; Funding acquisition; Investigation; Project administration; Resources; Supervision; Writing‐review & editing. **Michael L. Dustin**Conceptualization; Funding acquisition; Methodology; Project administration; Resources; Supervision; Writing‐original draft; Writing‐review & editing.

## CONFLICT OF INTEREST

Michael L. Dustin has filed a patent on supramolecular attack particles. Ranjeet Singh Mahla receives income from BMS in the form of a career development fellowship.

## Supporting information

Supporting Information

Supporting Information

Supporting Information

## Data Availability

Data available on request from the authors.
