# Correction to “Comparison of different methods for isolating CD8^+^ T lymphocyte‐derived extracellular vesicles and supramolecular attack particles”

**DOI:** 10.1002/jex2.83

**Published:** 2023-05-02

Jainarayanan, A. K., Capera, J., Céspedes, P. F., Conceição, M., Elanchezhian, M., Thomas, T., Bonner, S., Valvo, S., Kurz, E., Mahla, R. S., Berridge, G., Hester, S., Fischer, R., Dustin, L. B., Wood, M. J. A., & Dustin, M. L. (2023). Comparison of different methods for isolating CD8^+^ T lymphocyte‐derived extracellular vesicles and supramolecular attack particles. *Journal of Extracellular Biology*, 2, e74. https://doi.org/10.1002/jex2.74

In the originally published article, complete funding information was not included on the first page. The complete funding information is:

Funding Information

University of Oxford, Grant/Award Number: BB/M011224/1

Kennedy Trust for Rheumatology Research: KENN 20 21 17

European Commission Horizon 2020: ERC‐2021‐SyG_951329 and ERC‐2014‐AdG_670930

Wellcome Trust: 100262/Z/12/Z and 224343/Z/21/Z

We apologize for this error.

