# Supplementary material for: Comparison of different methods for isolating CD8+ T lymphocyte‐derived extracellular vesicles and supramolecular attack particles
Source: J Extracell Biol. 2023 Mar 13;2(3):e74. doi: 10.1002/jex2.74 (PMC11080737; doi:10.1002/jex2.74)
Supplement: Supplementary file 3 — Supporting Information [file JEX2-2-e74-s001.pptx]

## Slide 1
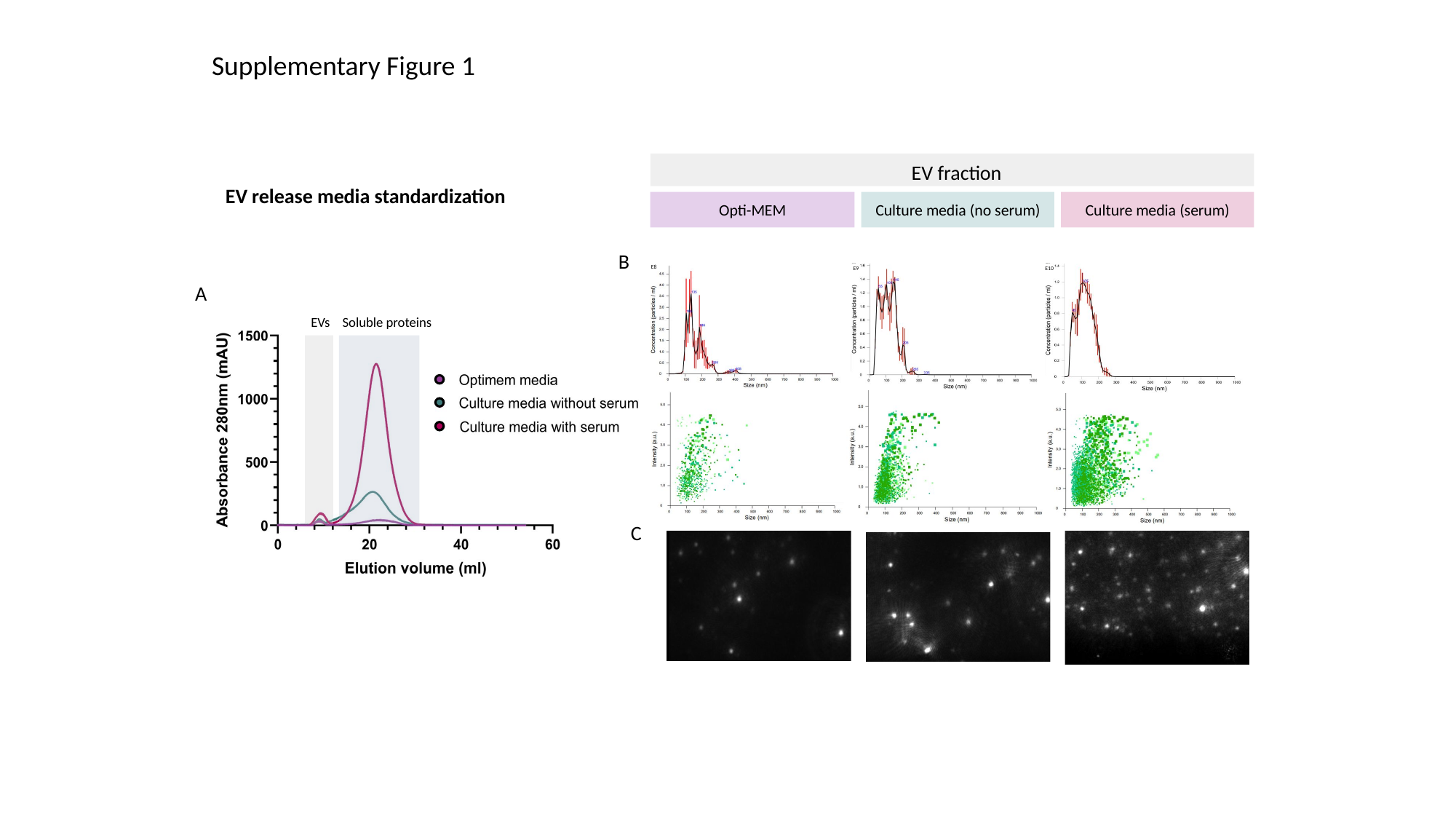

Supplementary Figure 1
EV fraction
Opti-MEM
Culture media (serum)
Culture media (no serum)
EV release media standardization
B
A
Protein aggregates
EVs
Soluble proteins
C
E8
E10
E9

## Slide 2
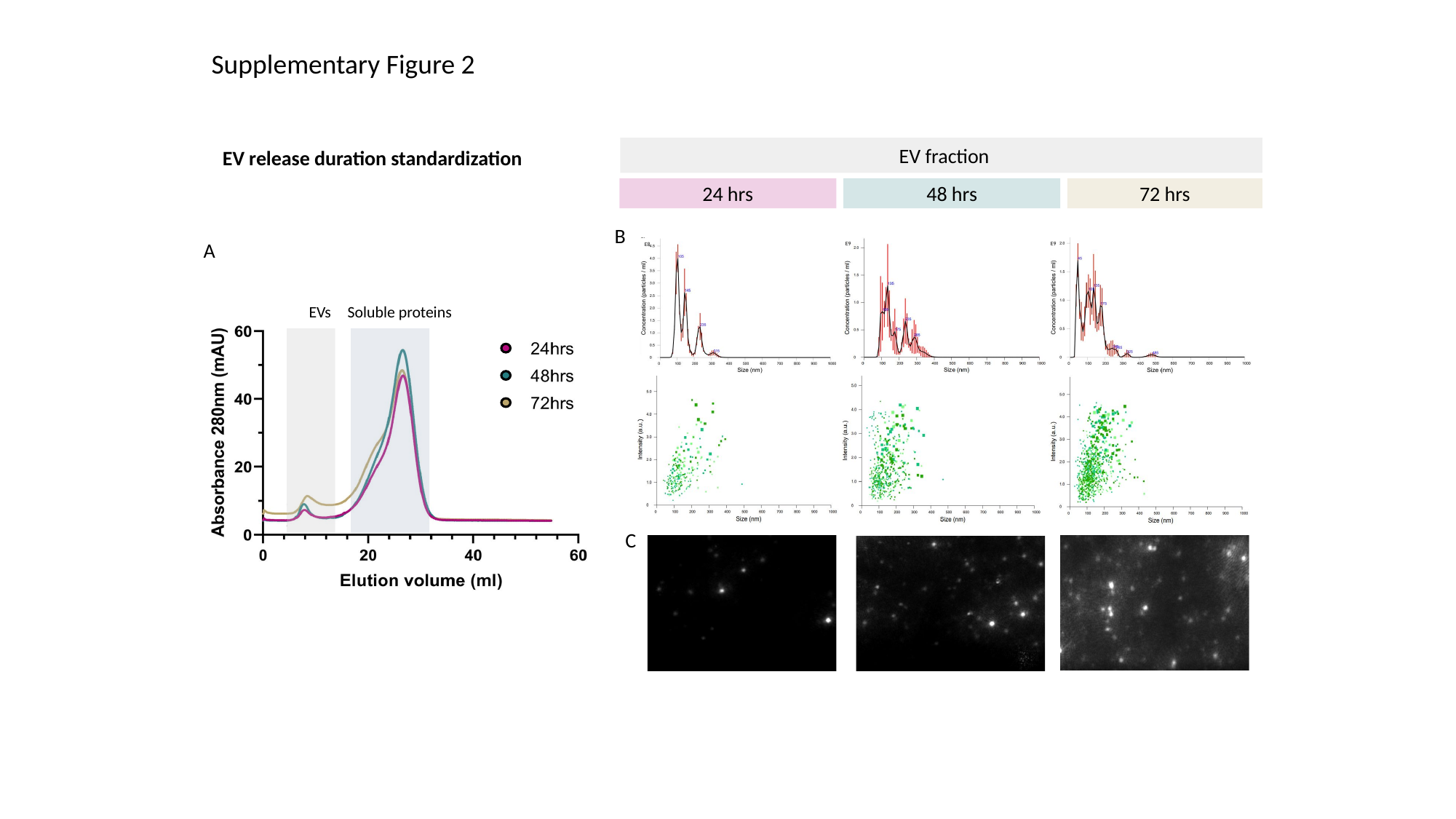

Supplementary Figure 2
EV fraction
24 hrs
48 hrs
72 hrs
EV release duration standardization
B
A
EVs
Protein aggregates
Soluble proteins
C
E9
E9
E8

## Slide 3
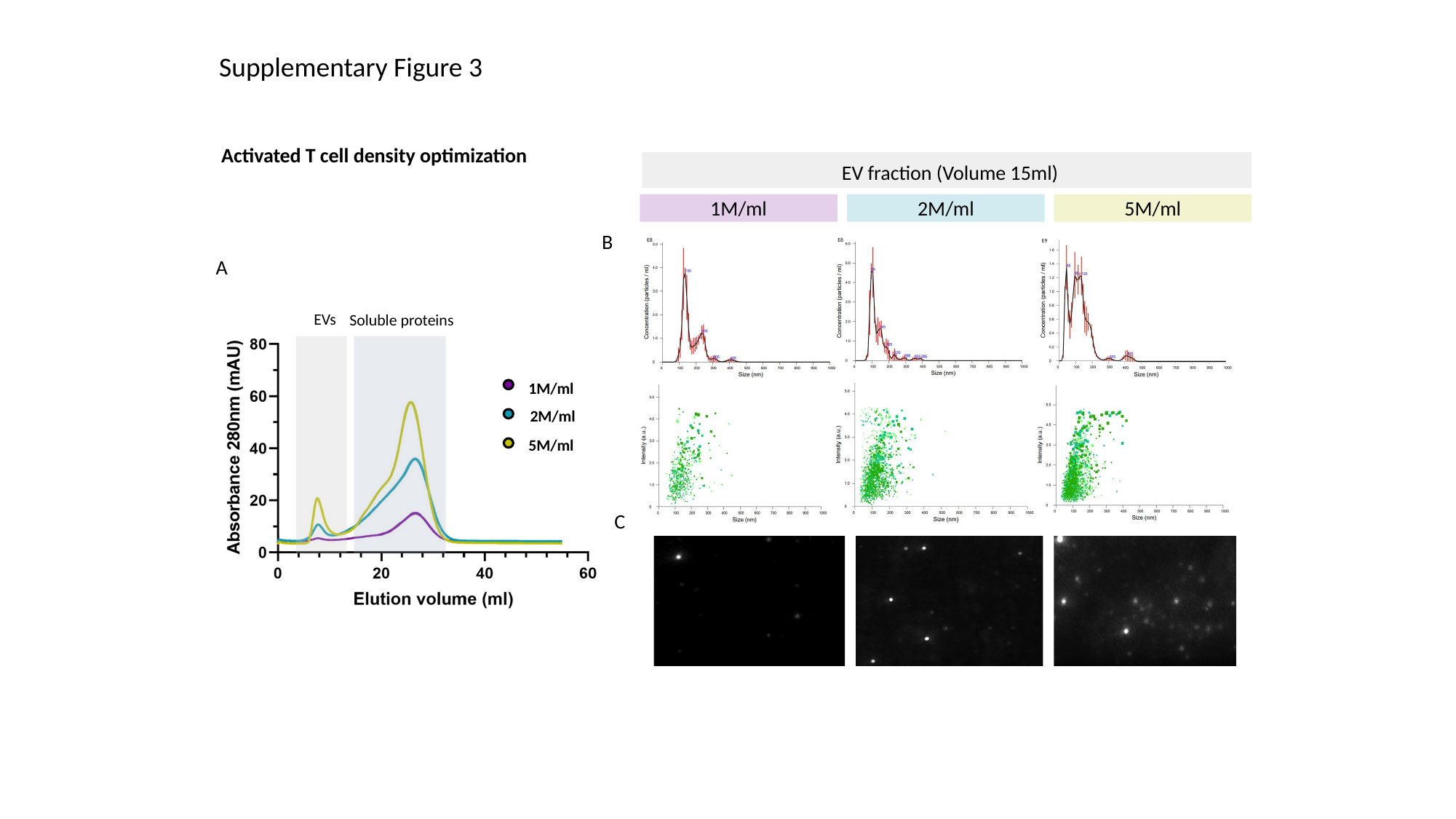

Supplementary Figure 3
Activated T cell density optimization
EV fraction (Volume 15ml)
1M/ml
2M/ml
5M/ml
B
E8
E8
E9
A
EVs
Protein aggregates
10M
30M
50M
1M/ml
2M/ml
5M/ml
Soluble proteins
C

## Slide 4
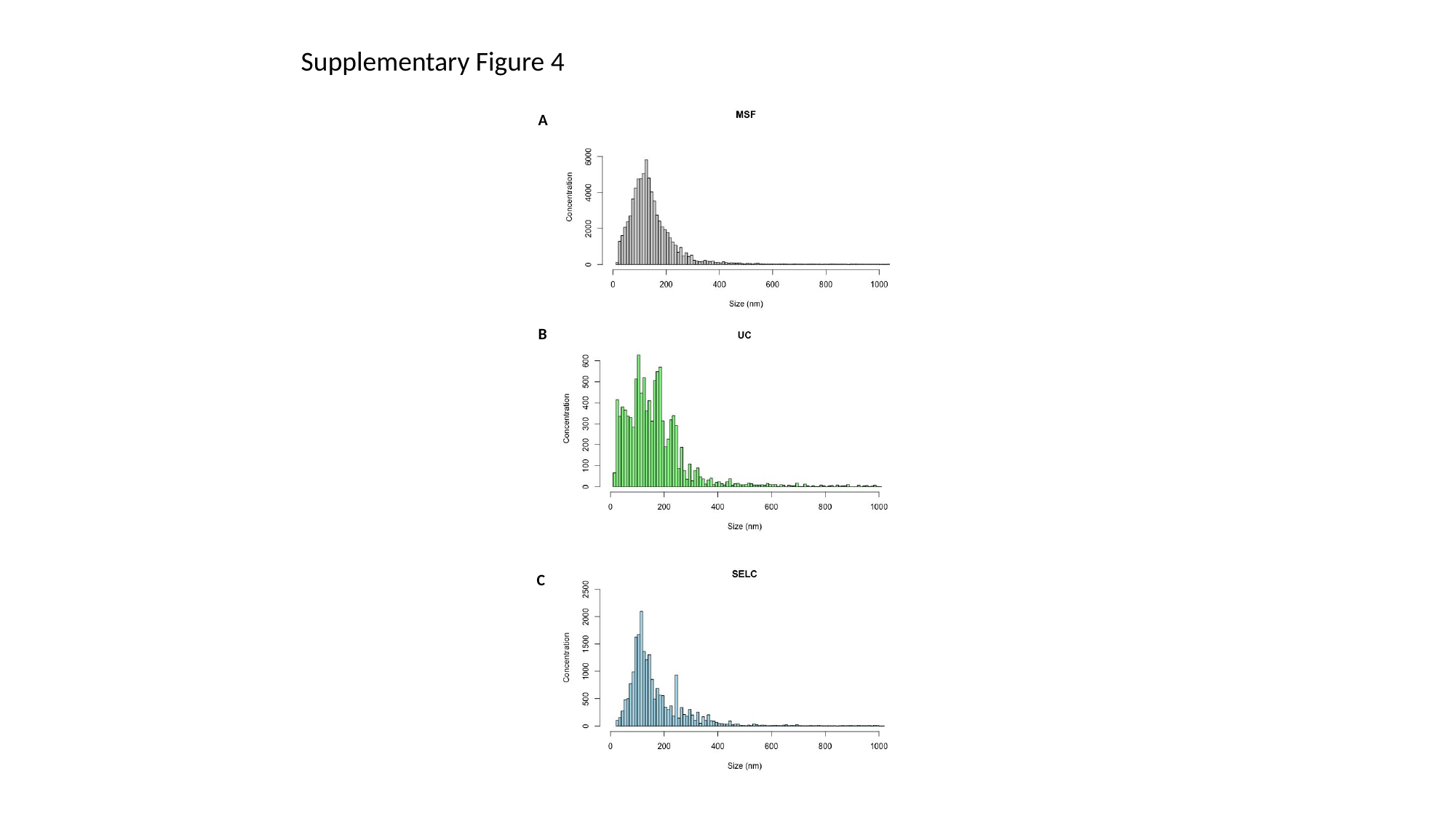

Supplementary Figure 4
A
B
C

## Slide 5
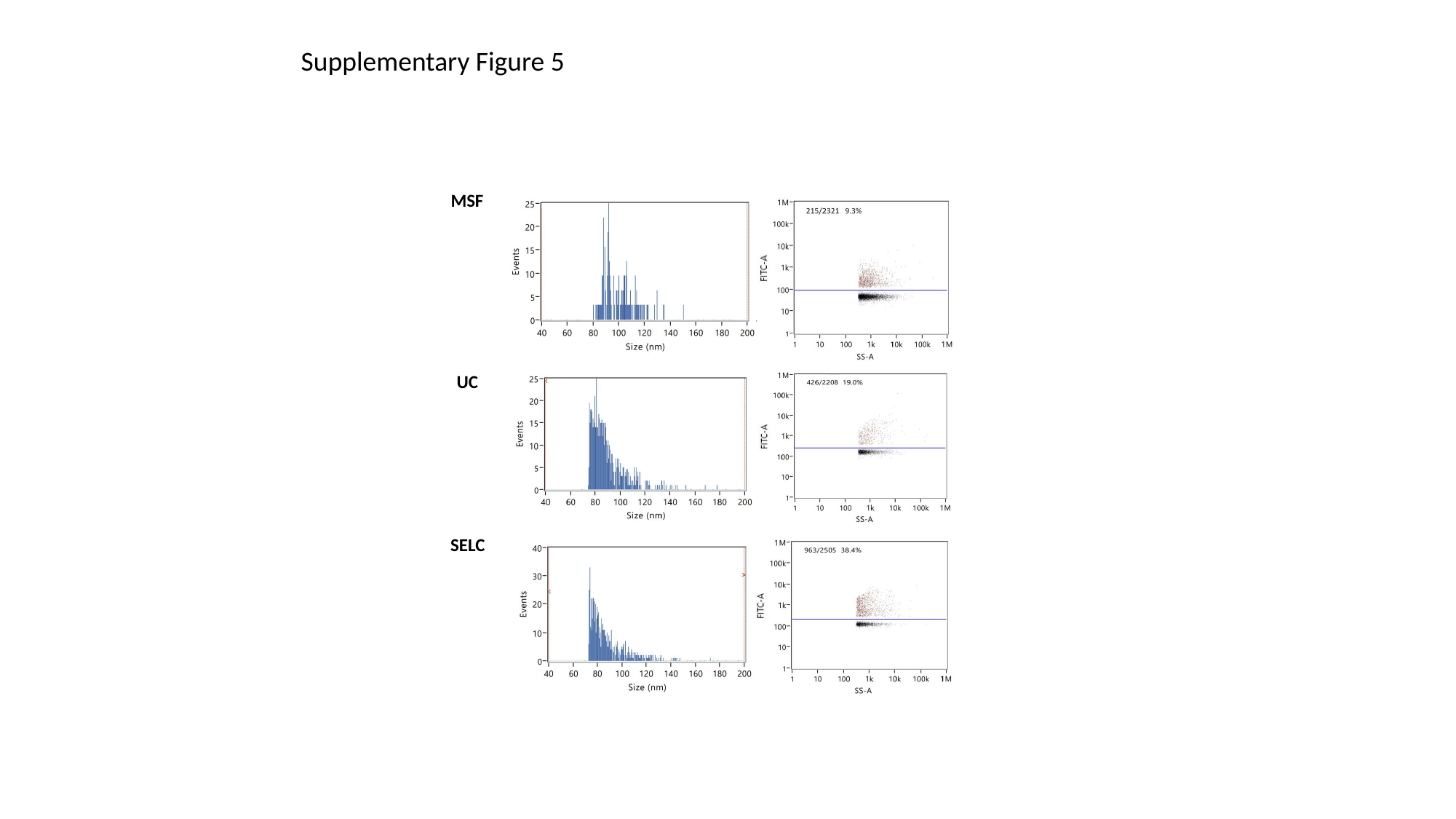

Supplementary Figure 5
MSF
UC
SELC

## Slide 6
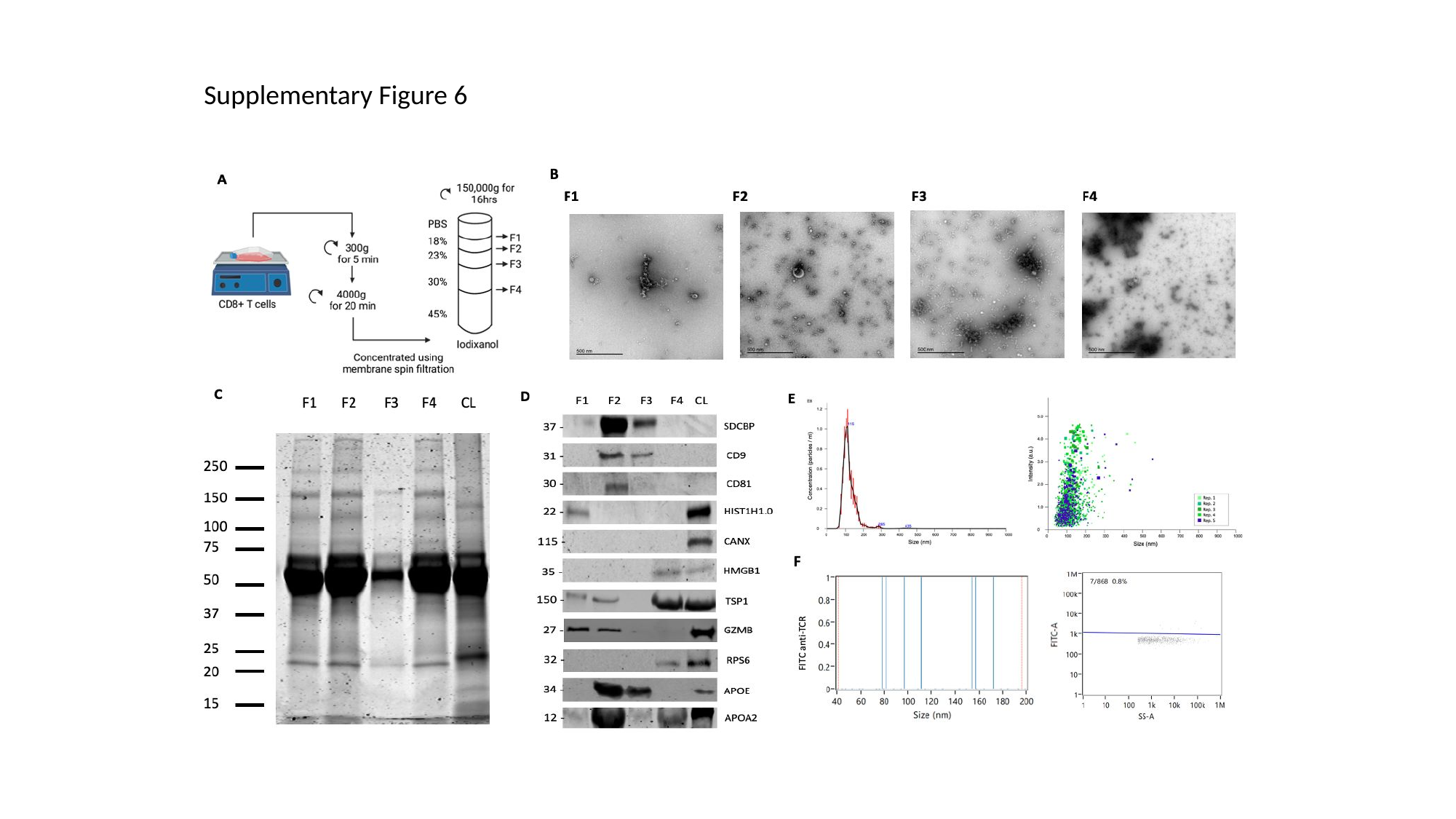

Supplementary Figure 6

## Slide 7
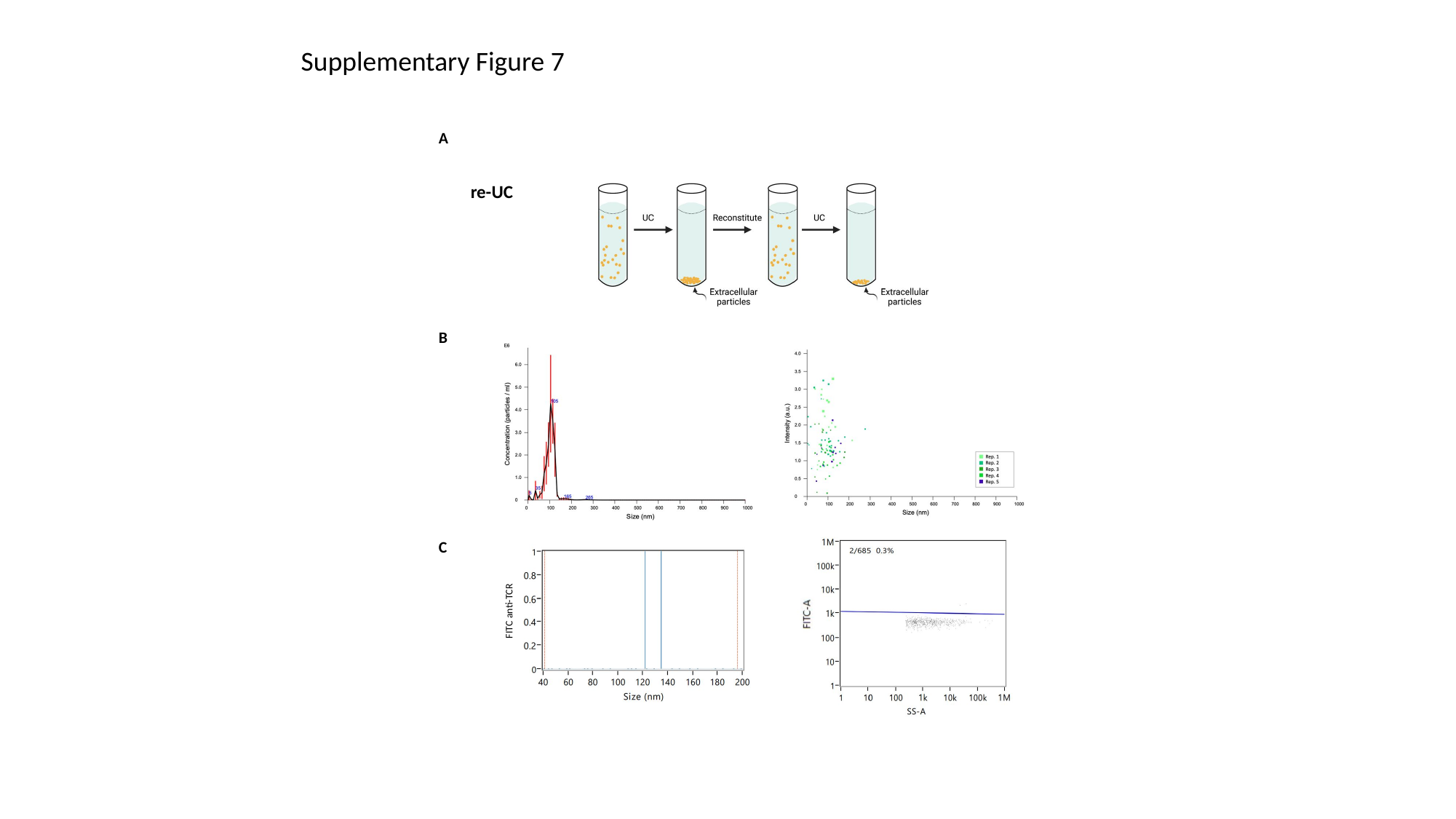

Supplementary Figure 7
A
re-UC
B
C
FITC anti-TCR

## Slide 8
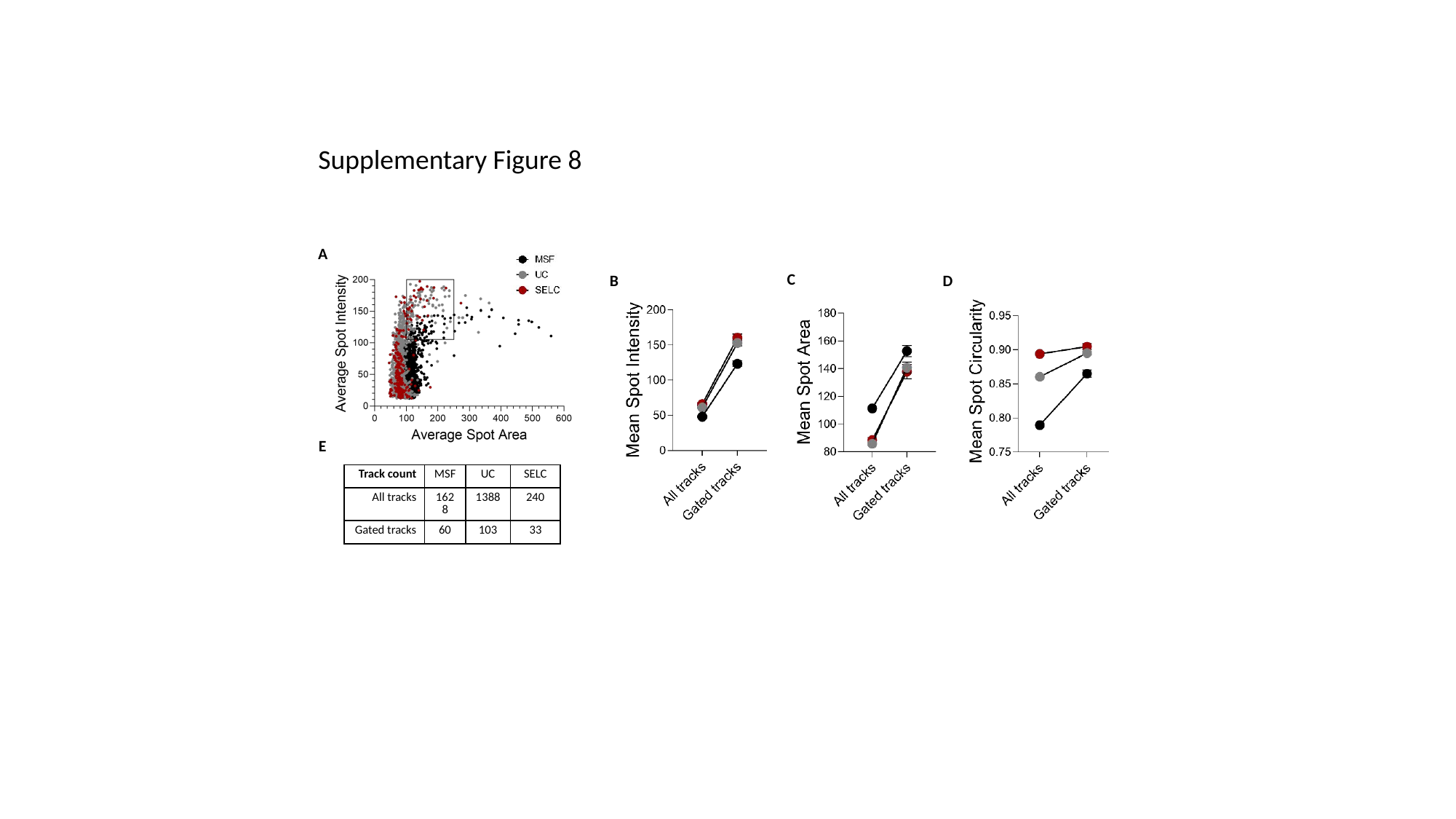

Supplementary Figure 8
A
A
C
B
D
E
B
| Track count | MSF | UC | SELC |
| --- | --- | --- | --- |
| All tracks | 1628 | 1388 | 240 |
| Gated tracks | 60 | 103 | 33 |

## Slide 9
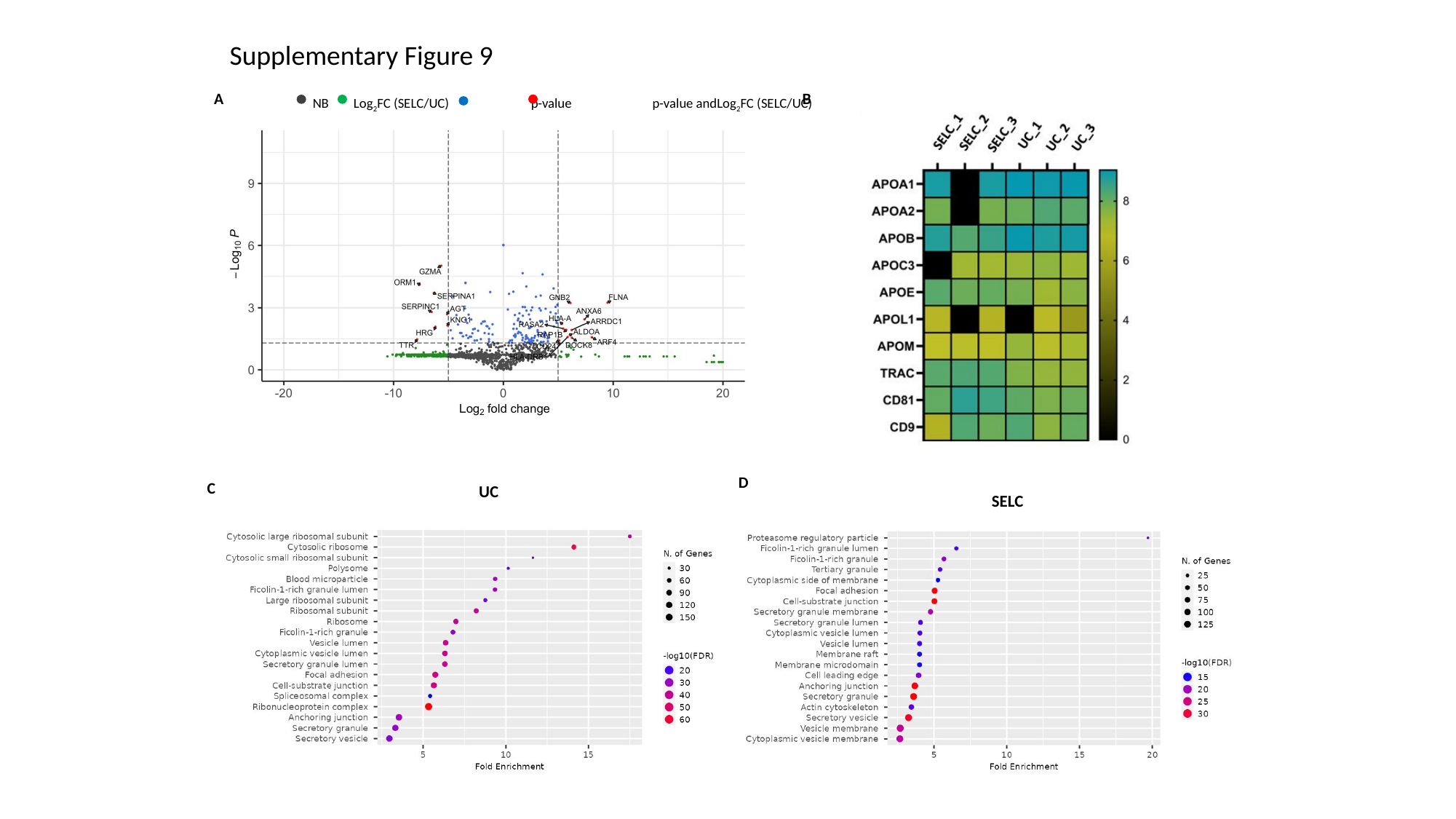

Supplementary Figure 9
NB Log2FC (SELC/UC)	p-value	 p-value andLog2FC (SELC/UC)
A
B
D
C
UC
SELC
